# Genome‐wide selection footprints and deleterious variations in young Asian allotetraploid rapeseed

**DOI:** 10.1111/pbi.13115

**Published:** 2019-04-17

**Authors:** Jun Zou, Lingfeng Mao, Jie Qiu, Meng Wang, Lei Jia, Dongya Wu, Zhesi He, Meihong Chen, Yifei Shen, Enhui Shen, Yongji Huang, Ruiyuan Li, Dandan Hu, Lei Shi, Kai Wang, Qianhao Zhu, Chuyu Ye, Ian Bancroft, Graham J. King, Jinling Meng, Longjiang Fan

**Affiliations:** ^1^ National Key Laboratory of Crop Genetic Improvement Huazhong Agricultural University Wuhan China; ^2^ Institute of Crop Sciences & Institute of Bioinformatics Zhejiang University Hangzhou China; ^3^ Department of Biology York University Heslington UK; ^4^ Center for Genomics and Biotechnology Haixia Institute of Science and Technology (HIST) Fujian Agriculture and Forestry University Fuzhou China; ^5^ CSIRO Agriculture and Food Canberra Australia; ^6^ Southern Cross Plant Science Southern Cross University Lismore NSW Australia

**Keywords:** allopolyploid, selection footprints, deleterious variations, introgression, Asian rapeseed

## Abstract

*Brassica napus* (AACC, 2*n* = 38) is an important oilseed crop grown worldwide. However, little is known about the population evolution of this species, the genomic difference between its major genetic groups, such as European and Asian rapeseed, and the impacts of historical large‐scale introgression events on this young tetraploid. In this study, we reported the *de novo* assembly of the genome sequences of an Asian rapeseed (*B. napus*), Ningyou 7, and its four progenitors and compared these genomes with other available genomic data from diverse European and Asian cultivars. Our results showed that Asian rapeseed originally derived from European rapeseed but subsequently significantly diverged, with rapid genome differentiation after hybridization and intensive local selective breeding. The first historical introgression of *B. rapa* dramatically broadened the allelic pool but decreased the deleterious variations of Asian rapeseed. The second historical introgression of the double‐low traits of European rapeseed (canola) has reshaped Asian rapeseed into two groups (double‐low and double‐high), accompanied by an increase in genetic load in the double‐low group. This study demonstrates distinctive genomic footprints and deleterious SNP (single nucleotide polymorphism) variants for local adaptation by recent intra‐ and interspecies introgression events and provides novel insights for understanding the rapid genome evolution of a young allopolyploid crop.

## Introduction

*Brassica napus* L. (A^n^A^n^C^n^C^n^, 2*n* = 38) is the third largest source of vegetable oil globally and plays an important role in increasing the productivity of wheat–rapeseed intercropping systems (Ebrahimi *et al*., 2017). *B. napus* is a young allopolyploid species derived from an interspecific cross between the two diploid progenitors, *B. rapa* (A^r^A^r^, 2*n* = 20) and *B. oleracea* (C^o^C^o^, 2*n* = 18). A genome‐based estimation has suggested a short history of post‐Neolithic speciation (~7500 years) and domestication (~700 years) of the species (Chalhoub *et al*., 2014). According to their geographic origin and seasonal crop type, *B. napus* oilseed cultivars were classified into several distinct genetic groups, including European winter, Asian semi‐winter, Canadian, Australian and European spring. In China, in the 1950s, *B. napus* replaced *B. rapa*, the local oilseed crop that had been cultivated for more than a thousand years (Li, 2001). ‘Shengliyoucai’ (SL), a collective name for multiple cultivars and their descendants, was the initial type of *B. napus* cultivated in China (Liu, 2000). Many distinct cultivars (e.g. Ningyou 7) were bred based on SL by introgression of traits from *B. rapa*, which has been frequently adopted to broaden the genetic diversity and improve the local adaptation of semi‐winter *B. napus* in Asia (Zhang *et al*., 2014). As a result, many Asian *B. napus* cultivars carry a common lineage derived from SL and/or *B. rapa*. In the 1970s, Asian rapeseed was further significantly improved by introgression of the double‐low traits (low seed glucosinolate and low erucic acid), which had first been discovered in Canada and then introduced into European rapeseed.

The ‘cost of domestication’ has been investigated in recent years, which suggests that the overall fitness of domesticated crops can be reduced as a result of the accumulation of deleterious genetic variants in their genomes (Moyers *et al*., 2018). Deleterious alleles are generally retained at a low frequency and tend to be purged by purifying selection (Fu *et al*., 2014). Several evolutionary factors have been reported to contribute to the number and frequency of deleterious variants in a domesticated population, such as demography, genetic drift and artificial selection (Gaut *et al*., 2018). More specifically, after the genetic bottleneck of domestication, deleterious variants remaining in the founder population tend to increase to a higher frequency because the purifying selection is less efficacious in a population with a smaller effective population size (Fu *et al*., 2014). In addition, genetic hitchhiking during artificial selection may increase deleterious variants to high frequency (Chun and Fay, 2011; Zhou *et al*., 2017). However, the fate of deleterious variants influenced by introgression is largely unexplored and, to our knowledge, examined in only a maize study (Wang *et al*., 2017a). Natural introgression, or artificial hybridization, is very common and plays crucial roles in the evolution history of crops. It is thus imperative to unveil how introgression shapes deleterious alleles, which could potentially have practical implications for crop breeding and improvement. Asian rapeseed experienced inter‐ and intraspecific introgression events during recent breeding history providing a unique opportunity for addressing the question.

**Figure 1:**
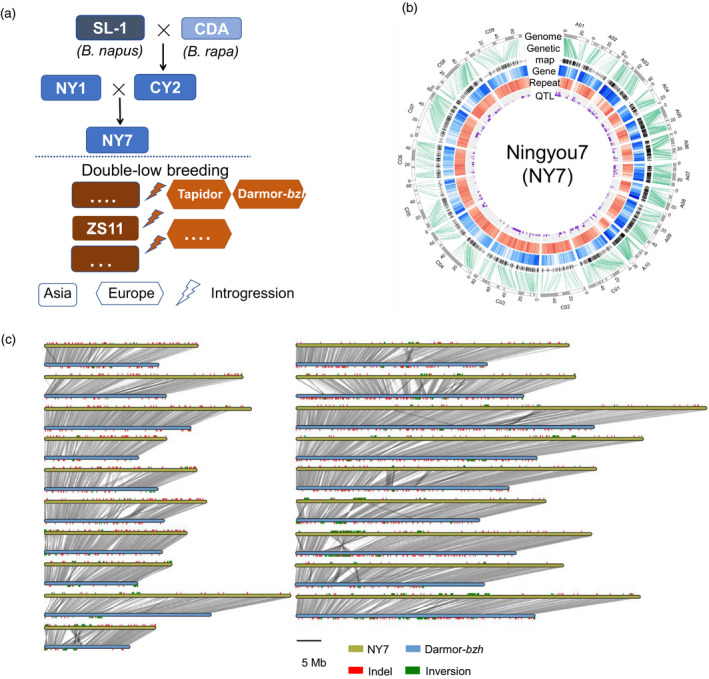
Pedigree and the genome assembly of NY7. (a) Asian rapeseed breeding history with the NY7 pedigree. Asian rapeseed experienced *B. rapa* introgression during the double‐high breeding stage (top panel) and then European rapeseed introgression during the double‐low breeding stage (low panel). In the low panel, only the three *B. napus* accessions (Tapidor, Darmor‐*bzh* and ZS11), which have been *de novo* sequenced, are shown. SL‐1: Shengliyoucai‐1; CDA: Chengduaiyoucai; CY2: Chuanyou2; NY1: Ningyou1; NY7: Ningyou 7; ZS11: Zhongshuang11. (b) Circos plot of the NY7 genome assembly with a genetic map, annotation and QTL. Circles inwards: pseudo‐chromosomes (black or white bars represent individual scaffolds), genetic maps, gene density, repeat density and QTL based on the Tapidor×NY7 population (*Bna* TNDH). (c) Genomic synteny and variations between the two *de novo* assembly genomes, NY7 and Darmor‐*bzh*.

In this study, we sequenced and *de novo* assembled the genome of Ningyou7 (NY7), an elite Chinese semi‐winter *B. napus* cultivar developed from early SL cultivars with introgressions from local diploid rapeseed *B. rapa* but without introgression of the double‐low traits from European rapeseed (Luo *et al*., 2017; Qiu *et al*., 2006) (Figure 1a). This result contrasts with the recently sequenced Asian cultivar ZS11, which carries double‐low alleles (Sun *et al*., 2017). Dissecting the genome of NY7 provides the opportunity to understand the evolutionary footprints associated with local adaptation and early artificial selection of Asian rapeseed. Furthermore, the genomes of the parental lines of NY7 were also *de novo* assembled, and a comprehensive genomic comparison was undertaken among NY7 and its parental lines as well as other publicly available genome sequences of a diverse group of European and Asian rapeseed cultivars (Huang *et al*., 2013; Schmutzer *et al*., 2015; Shen *et al*., 2017; Wang *et al*., 2018). The comparison of *B. napus* genomes helped us trace the origins of valuable adaptive alleles, genomic structural variations and deleterious variations during the recent breeding history of Asian rapeseed and provided novel insights into the significant impacts of inter‐ and intraspecific introgressions arising from local selective breeding on a young allopolyploid species.

## Results

### *De novo* genome assemblies of NY7 and its parental lines

We sequenced and assembled the genome of the Asian *B. napus* cultivar NY7 using a combination of three technologies (Table 1; for details, see Table S1). The assembled genome based on Illumina and PacBio data was confirmed by the syntenic relationship of an updated ‘Tapidor×NY7’ (*Bna*TNDH) genetic linkage map (Long *et al*., 2011) constructed using 353 lines with a total of 2304 backbone markers (Table S2). The resulting assembly spans 994 Mb, covering ~85% of the NY7 genome (1170 Mb, Figure S1) based on *K*‐mer estimation. The assembly was further improved by three‐dimensional (3D) chromosome conformation capture sequencing (HiC), which increased the scaffold N50 to 6.90 Mb (Table 1 and Figure S2). Based on this assembly, we generated 19 chromosomal pseudo‐molecules anchored to the updated *Bna*TNDH genetic linkage map and two other published genetic linkage maps (Chalhoub *et al*., 2014; Delourme *et al*., 2013) (Figure S3). In total, 372 scaffolds were anchored to the linkage maps, representing 890 Mb (89.5%) of the total assembly length (Figure 1b; Table 1, Figure S4), providing a more comprehensive genome coverage than those previously published for Darmor‐*bzh* and ZS11 (Table 1).

**Table 1:** Summary of genome assembly and annotation of Asian rapeseed NY7 and its parental lines

NY7				
Sequencing	Insert libraries	Illumina	PacBio RS II	HiC
	160 bp‐20 Kb	~200×	~30×	~100×
Scaffolds	N50 size (Mb)	L50	The maximum (Mb)	Total non‐N size (Mb)
	1.27	229	7.41	956.9
Super‐scaffolds by HiC	6.91	37	33.78	956.9
Chromosomes by two genetic maps	Total anchored/non‐N size (Mb)	Unique marker No.	Darmor‐*bzh* c Anchored/non‐N size	ZS11^c^Anchored/non‐N size
	892.0/874.0	13,164	645.4/553.4	855.0/797.7
Annotation	Gene models	BUCSOs/CEGMA (%)	Supported by EST and Swiss‐protein, etc. (%)	Repetitive elements (%)
	104,179	98.5/98.4	97.0	45.0

Parental lines in the NY7 pedigree				
Parental lines^a^	300 bp‐10 Kb Illumina data (Gb)^b^	*K*‐mer estimate size (Gb)	Genome assembly (Mb)	Scaffold N50 size (Kb)
SL‐1	147.4	1.175	860.7	331.0
CDA	106.6	0.514	412.2	178.9
NY1	153.0	1.221	845.7	369.6
CY2	118.1	1.178	869.5	293.2

a For details, see Figure 1a.

b including the libraries with 3 and 10 Kb insertion sizes.

c Comparison of assemblies indicates that while NY7 included only 2% (18 Mb) of ‘N’, Darmor‐*bzh* and ZS11 included 14% (92 Mb) and 7% (57 Mb), respectively.

To evaluate the quality of the NY7 genome assembly, 643 944 publicly available *B. napus* expressed sequence tags (ESTs) from GenBank were mapped to the genome, of which ~97% could be aligned with an average identity of 97%. We further examined the assembly based on the congruence of transcriptomes of 47 lines of the *Bna*TNDH mapping population and built genome‐ordered graphical genotypes (GOGGs) (Table S3). This approach allowed us to include an additional ~1.3 Mb (two scaffolds) sequence and to correct 52 mis‐assemblies with a cumulative length of ~15.9 Mb (for details, see Methods; Table S3).

As found in other *B. napus* genome assemblies, the NY7 genome includes a number of extensive repetitive regions in which no or few molecular markers could be found (Figure 1b). However, by using Hi‐C sequence data (Figure S5) and centromeric‐specific markers from the *Bna*TNDH genetic map (Long *et al*., 2011), we were able to extend the end of chromosome A03 in the NY7 assembly by ~3.7 Mb (38.3~42.0 Mb), a result that was not achieved in the three previously sequenced *B. napus* genomes (Bayer *et al*., 2017; Chalhoub *et al*., 2014; Sun *et al*., 2017). These genomic regions were not found in the anchored Darmor‐*bzh* pseudo‐chromosomes but in an unanchored scaffold that was assigned to chromosome A03 of Darmor‐*bzh* and *B. rapa* cv. Chiifu‐401 v2.5 (Wang *et al*., 2011).

A total of 104 179 protein‐coding genes were annotated in the NY7 genome (Table 1). The NY7 assembly appears to cover the majority of the gene space (Table S4) based on the evidence of 98.5% and 98.4% genes of the CEG and the plantae BUSCO data sets (V3) (Simao *et al*., 2015), respectively, having a match in the NY7 genome. Repetitive sequences accounted for 44.7% of the NY7 genome assembly (Table 1), with 16.2% of the genome represented by long terminal retrotransposons.

To understand the inheritance pattern contributing to NY7, we also sequenced four parental lines (SL‐1, CDA, NY1 and CY2) of NY7, including the founder landrace SL‐1 and the *B. rapa* accession CDA (Figure 1a). Drafts of the four genomes were *de novo* assembled by deep sequencing with libraries of 3–10 Kb insertion sizes (Table S1). The final genome assemblies (average scaffold N50: 300 Kb) covered 70%~80% of the estimated genome sizes (Table 1). Moreover, we also updated the genome of Tapidor, another parental line of the *Bna*TNDH population, with more genomic sequencing data (Table S1). Based on the same assembly pipeline as that used for NY7, we were able to increase the assembly size of Tapidor to 1.02 Gb with a scaffold N50 size of 806 Kb and further anchored 853 Mb sequences to the *Bna*TNDH linkage map (Figure S5). The old version of the Tapidor genome had an assembly size of 0.63 Gb and an N50 of 197 Kb (Bayer *et al*., 2017).

### Structural variations between Asian and European cultivars based on four *de novo* genome assemblies

We carried out pairwise genome comparisons between NY7 and each of the two sequenced European cultivars (Darmor‐*bzh* and Tapidor) and the Asian cultivar (ZS11), all three carrying the introgressed double‐low alleles. Between NY7 and Darmor‐*bzh*, 1.49 million SNPs and 0.17 million small insertions/deletions (Indels) were observed. Although these two cultivars have a high level of synteny, frequent small and large structural variations were observed (Figure 1c and Figure S5). Compared to the NY7 genome, the Darmor‐*bzh* genome had 42.9k insertions covering 118.3 Mb and 82.3k deletions covering 162.1 Mb (Table S5 and Figure 1c). Other structural variations included 34.5 k of inversion events covering 45.1 Mb (26 bp~100 kb in size) and 4.4 k of translocation events covering 9.4 Mb (>1 kb in size). Similarly, we also found many genomic variations between NY7 and the other two sequenced cultivars, Tapidor and ZS11 (Table S5). For example, we found a 3.9 Mb deletion in Tapidor's C03 compared to NY7 (chrC03: 0.7~4.6 Mb) based on their genomic synteny (Figure S6). Interestingly, the region overlapped with a QTL (*es. C3‐3*, chrC03: 0.07~2.3 Mb) associated with flowering time and seed yield. The detailed divergent/syntenic regions between Tapidor and NY7, together with the QTL of the ‘Tapidor×NY7’ DH population (i.e. *Bna*TNDH), can be visualized in the ‘BnPedigome’ database generated in this study ( http://ibi.zju.edu.cn/bnpedigome/).

### Phylogeny and genomic signatures of Asian rapeseed

#### Genetic diversity and phylogenetic tree of Asian and European rapeseeds

**Figure 2:**
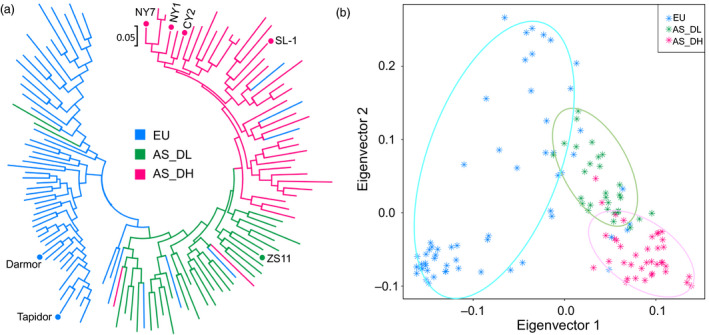
Genetic divergence of Asian and European rapeseeds. (a) Phylogenetic tree and (b) principal component analysis of Asian (AS) and European (EU) rapeseeds. Asian rapeseeds were separated into two groups: double‐high (AS_DH) and double‐low (AS_DL). The *de novo* sequenced cultivars are indicated by dots.

To analyse the genetic differentiation of Asian *B. napus* cultivars from European cultivars, in addition to the genomic data generated in this study, we collected publicly available genomic data of 68 Asian (30 double‐low and 38 double‐high) (Chalhoub *et al*., 2014; Huang *et al*., 2013; Shen *et al*., 2017; Wang *et al*., 2018) and 59 European accessions (Chalhoub *et al*., 2014; Schmutzer *et al*., 2015; Wang *et al*., 2018) (Table S6). We calculated whole‐genome genetic diversity (π) for each of the three groups, that is European (EU; including both double‐low and double‐high accessions), Asian double‐low (AS_DL) and Asian double‐high (AS_DH). The EU group had the highest genetic diversity (1.45e‐3), followed by AS_DL (1.41e‐3) and AS_DH (1.19e‐3). For the two subgenomes, the C subgenome had a higher nucleotide diversity in European accessions (1.25e‐3) than in Asian accessions (1.02e‐3); the A subgenome had the highest diversity in AS_DL (1.89e‐3), followed by AS_DH (1.79e‐3) and EU (1.78e‐3), even though the difference was not significant (Figure S8). The LD analysis indicated that the Asian rapeseeds had a slower LD decay rate than the European rapeseeds in both the A and C subgenomes (Figure S9). Phylogenetic and principal component analyses (PCA) (Figure 2 and Figure S7) showed two distinct independent branches representing the Europe and Asian lineages. While the AS_DL accessions were clearly separated from the AS_DH accessions, the former was genetically closer to the EU accessions (Figure 2). These results suggest that the Asian accessions have been reshaped to form a new domesticated ecotype, distinct from European accessions, by strong artificial selection in a relatively short period of time.

#### Demographic origins of Asian rapeseeds

**Figure 3:**
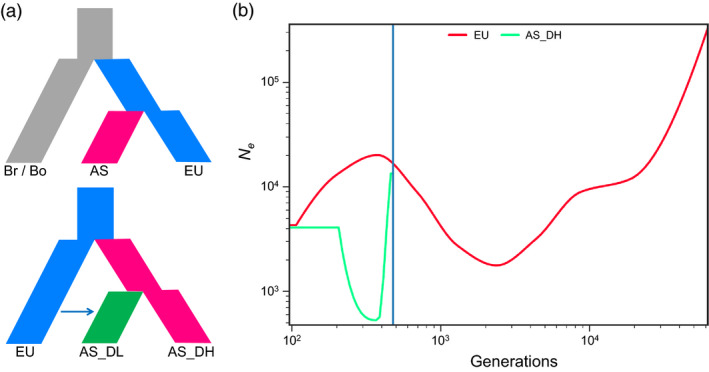
Demographic inference for Asian rapeseeds. (a) The best demographic models for the origin of Asian (AS) and its double‐high (AS_DH) and double‐low (AS_DL) varieties. EU, Br and Bo refer to European rapeseed and its two progenitors, *B. rapa* and *B. oleracea*, respectively. (b) Divergent time (generation) and effective population size (*N*e) changes for the European (EU) and Asian double‐high (AS_DH) rapeseed populations estimated using SMC++.

To determine the origin of Asian rapeseeds, we carried out demographic analysis based on the available SNP data from accessions of the two diploid progenitor species (34 *B. rapa* and 37 *B. oleracea*) (Cheng *et al*., 2016). The analyses were performed using four different demographic simulation models based on putative neutral SNPs in intergenic regions (Figure S10; for details, see Methods). Demographic analyses based on both the A and C subgenomes supported the model in which European rapeseed first diverged from its ancestor *B. rapa* and subsequently bifurcated to generate Asian rapeseed (Figure 3a and Table S7), that is supporting a European origin of the Asian rapeseeds, consistent with historic records (Liu, 2000). We further estimated the time when the Asian rapeseeds diverge from the European ones using SMC++ (Terhorst *et al*., 2017). Based on a substitute rate of 9e‐9 (Qi *et al*., 2017), we found that Asian rapeseeds appear to have split from European rapeseeds within a period of 500 years (Figure 3b), with a notable decrease in population size representing a genetic bottleneck. Furthermore, we used the same demographic analysis approach to test the models for the origin of AS_DL rapeseeds (Figure S11). We found evidence for the hybridization origin model; that is, AS_DL first split from AS_DH, and then, introgression from European double‐low rapeseeds occurred (Figure 3a and Table S7).

#### Genomic signatures of Asian rapeseeds under local selection

**Figure 4:**
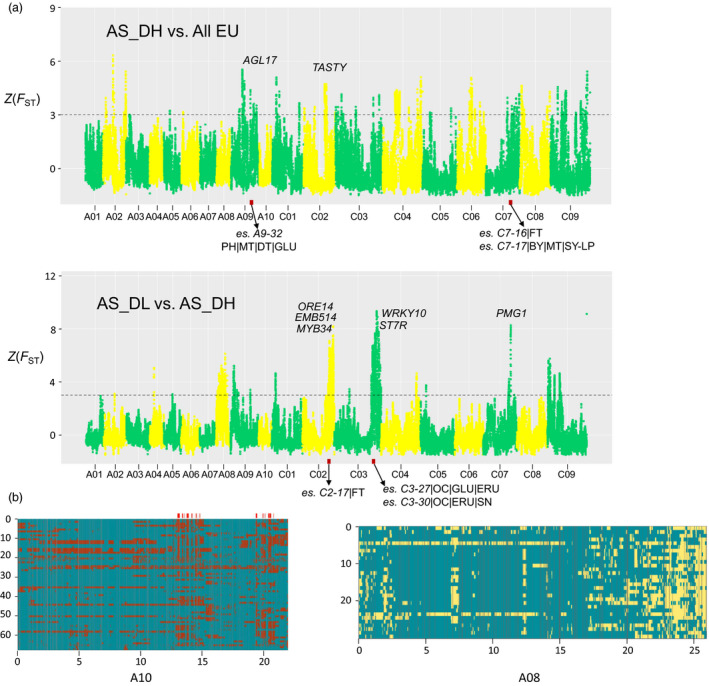
Genomic selection signals in Asian rapeseeds. (a) Significantly divergent genomic regions of Asian double‐high rapeseeds (AS_DH) relative to the European rapeseeds (EU) are shown on the top of the subfigure, and significant divergent genomic regions between Asian double‐low rapeseeds (AS_DL) and AS_DH are shown at the bottom. Selected genes and QTL found previously in those regions are indicated. (b) Local ancestry inference for Asian rapeseed. Left: Local ancestry inference for the 68 Asian rapeseeds in chromosome A10 (red: *B. rapa* genetic background; blue: EU genetic background). The red bars on the top indicate that the introgression events from *B. rapa* into the Asian rapeseed population have been almost fixed (i.e. found in >90% samples). Right: Local ancestry inference for the 30 Asian double‐low rapeseeds in chromosome A08 (blue: EU genetic background; yellow: Asian double‐high genetic background). The *X*‐axis represents the genomic locations of each chromosome. The *Y*‐axis presents representative individuals used for the ancestry inference. For details of all chromosomes, see Figure S14.

We performed large‐scale genomic scans (*F*_ST_) between Asian and European rapeseeds to uncover the most differentiated genomic regions in the two groups, that is genomic signatures of the Asian rapeseeds associated with breeding processes. Between the AS_DH and EU groups, we found a total of 1665 highly differentiated scanning windows (Z(*F*_ST_) > 3), covering 28.3 Mb of the genome and containing 2519 genes (Figure 3). The genes were unequally distributed in the two subgenomes, with 23.1 and 5.2 Mb in the C and A subgenomes, respectively, suggesting an asymmetric selection pattern in the two subgenomes. A total of 303 QTL for seed yield have been mapped using the *Bna*TNDH population derived from Tapidor x NY7 (Luo *et al*., 2017). Of them, 13 QTL reside within those highly divergent regions (Table S8), for example a divergent region (40.0~40.8 Mb) in chromosome A09 overlapping with a QTL (*es. A9‐32*) associated with seed development and maturity. This region contains a *Brassica* orthologue of *AGL17‐2*, a gene controlling flowering time in Arabidopsis (Figure 4a). Other flowering time‐related QTL (*es. C7‐16*,* es. C7‐17*) could also be found within the genomic regions that are highly divergent between the European and Asian groups.

Highly divergent genomic selection signatures were also detected between the Asian double‐low and double‐high rapeseeds, covering 30.2 Mb genomic regions (A subgenome: 8.0 Mb; C subgenome: 22.2 Mb). Five Mb‐scale peaks found in chromosomes A08, A09, C02, C03 and C09 perfectly matched results from a previous GWAS (Wang *et al*., 2018). These regions contain genes associated with seed‐quality traits, including erucic acid content (EAC), glucosinolate content (GSC) and seed oil content (SOC). Two QTL (*es. C3‐27* and *es. C3‐30*) associated with these traits were located in the highly divergent region in chromosome C03 (Figure 4a). These genomic footprints provide insights for the improvement of Asian double‐low rapeseeds through breeding.

We further performed a local ancestry inference (LAI) analysis to trace the genomic changes during the inter‐ and intraspecific introgression progresses during the breeding history of Asian rapeseeds. Interestingly, there were extensive introgression signatures of *B. rapa* in Asian rapeseeds, with highly conserved regions (in >90% accessions) covering 8.8 Mb of the genome. These regions contain important allelic patterns maintained in the Asian rapeseed population, including the *Brassica* orthologue (in chromosome A10) of *FLC,* a well‐characterized gene responsible for the regulation of flowering time in Arabidopsis (Figure 4b and Table S9). There is strong evidence for the fixation of *B. rapa* alleles contributing to early flowering time, high erucic acid, *Sclerotinia* resistance, and yield traits in the gene pool of AS_DH (Table S9 and Figure S14a). In the AS_DL genome, there exist highly conserved introgression patterns from European accessions, notably those Mb‐scale regions on A08, A09 and C03. These regions contain genes associated with low glucosinolate and erucic acid that are prevalent in the modern rapeseed gene pool (Figure 4 and Figure S14b). These observations indicate that during the breeding process of double‐low cultivars, the genomic regions of Asian rapeseeds controlling double‐high traits have been subjected to ongoing introgression from European double‐low genetic backgrounds and selected for improved seed‐quality traits.

### Genomic inheritance of the NY7 pedigree accessions

**Figure 5:**
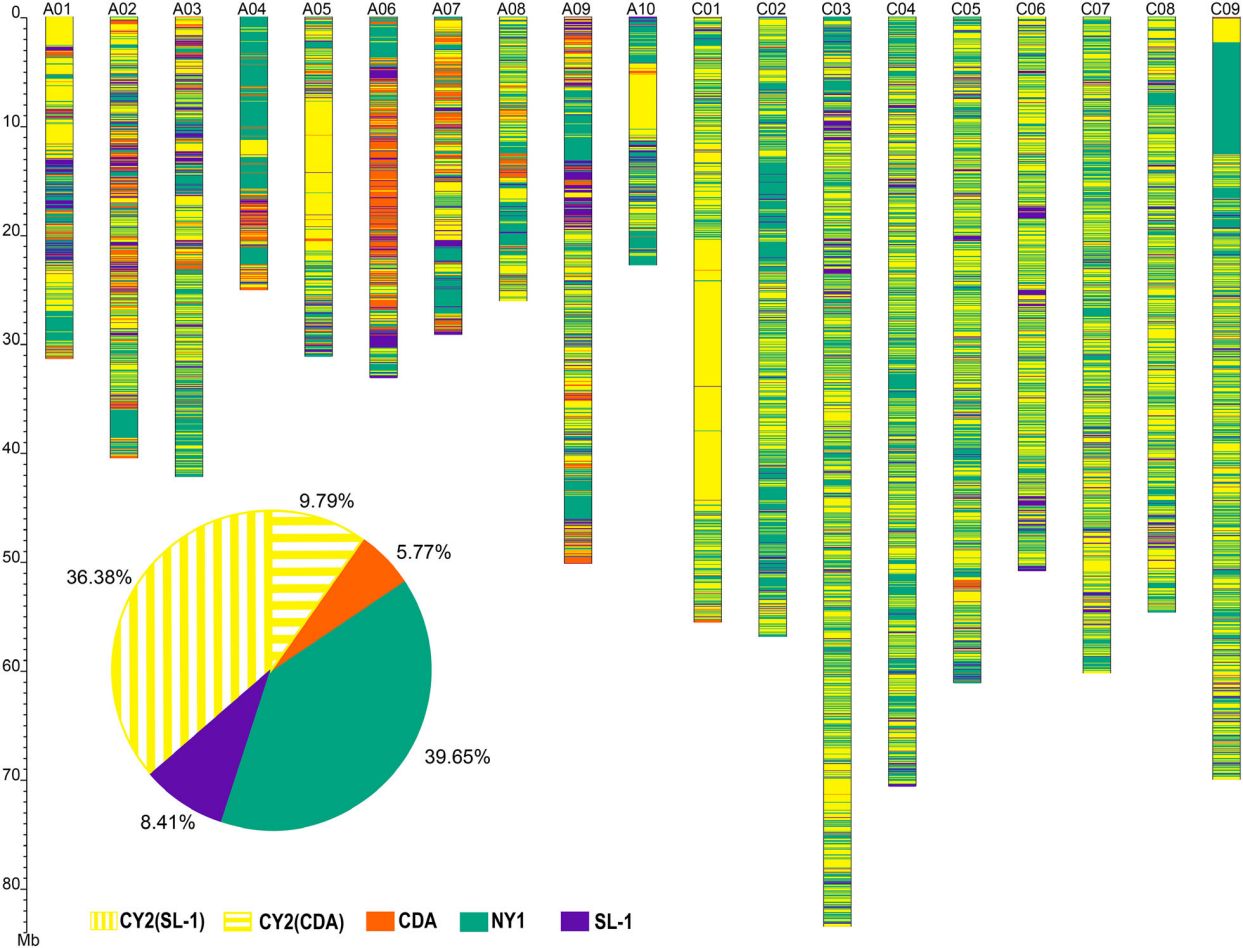
Identity‐by‐descent (IBD) inheritance pattern for the NY7 pedigree. For each chromosome, the genomic contributions by the four different parental lines to NY7 were colour‐coded as CY2 (yellow), CDA (orange), NY1 (green) and SL‐1 (purple). The accumulated percentages of genomic contribution by the four parental lines are shown in the pie graph. The genomic contribution of CY2 to NY7 was further divided into those from SL‐1 (36.38%) and CDA (9.79%).

To determine patterns of genomic inheritance, identity by descent (IBD) of NY7 was estimated by comparing the NY7 genome to the genomes of its four parental lines (Figures 1 and 5). As expected, the greatest overall genetic contributors to NY7 were its two direct parental lines NY1 (40%) and CY2 (46%) (Figures 1 and 5), consistent with previous results based on the SNP chip analysis (Wang *et al*., 2017b). We observed many Mb‐scale NY7 genomic blocks derived from NY1 or CY2. For example, an ~25 Mb region contributed by CY2 was found in the middle of chromosome C01 (from ~20 to ~45 Mb) of NY7 (Figure 5). Interestingly, this region seemed to be absent in NY1 because no assembled scaffolds or reads were found in the NY1 assembly and sequencing results, which might have been caused by homoeologous exchange between A01 and C01 in the progenitor NY1 genome (Figure S15), a phenomenon frequently observed in other studies (Chalhoub *et al*., 2014; Xiong *et al*., 2011). In addition, at the top of chromosome C09 of NY7 (0–12.6 Mb), we also observed large regions derived from CY2 (0–2.3 Mb) or from NY1 (2.3–12.6 Mb), and these may also be due to homologous exchanges occurred in the genomes of NY1 and CY2 (Figure S15). Meanwhile, a large proportion of the NY7 genomic segments were originated from the other two indirect progenitor parental lines SL‐1 (8.41%) and CDA (5.77%) (Figure 5; see Table S10 for a list of the detailed IBD regions with QTL information).

CDA is a semi‐winter Asian *B. rapa* rapeseed cultivar adapted to Asian production areas. We found that the ~6% NY7 genomic regions contributed by CDA (Figure 5) contained favourable QTL contributing to maturation time (*es. A1‐24*), oil content (*es. A4‐19*,* es. A6‐10*), seed weight (*es. A4‐4*) and disease resistance (*es. A4‐15*) (Table S10). Consistent with this finding, those regions also contained *Arabidopsis* orthologues with functions in the regulation of flowering time, such as *SEF*,* ELF8*,* REF6* and *VIP4* (Table S10). It is worth mentioning that the ~6% contribution of the CDA genome to NY7 could be underestimated, as a portion of the CDA contribution could have been considered to be from CY2, one of the two direct parents of NY7. To solve this issue, we further separated the contribution of CY2 into those from SL‐1 and CDA. In doing so, we found a further 9.79% contribution from CDA (Figure 5).

To further explore the contribution of CDA in the development of Asian *B. napus*, we first identified nonsynonymous SNPs in the *B. napus* population and then calculated the frequency of the SNPs representing CDA in the genes related to flowering time in the Asian and European groups. We found that the gene with the most differentiated allele frequency between the Asian (>0.80) and European (0.14) groups is chrA02 g004876 (Table S11), a homologue of Arabidopsis *HDA5* that has a potential role in regulating flowering time through histone modification (Luo *et al*., 2015). Notably, homologues of four other Arabidopsis flowering time‐related genes (*PRR5*,* HUA2*,* VIP4* and *CDF1*) were found in the flanking regions (A02: 37.8~39.1 Mb) of chrA02 g004876. All these genes had a higher allele frequency in the Asian group than in the European group; among these genes, *HUA2* was already fixed in the Asian group (Table S11). In addition to the significant changes in allele frequency, we also found a PAV (presence and absence variant) in a homologue of Arabidopsis *CDF2* (*CYCLING DOF FACTOR2*), which seems to be absent in SL‐1 (Figure S12). *CDF2* encodes a repressor of the transcription factor *CONSTANS* (*CO*), a key regulator of the photoperiodic flowering response in *Arabidopsis* (Imaizumi *et al*., 2005). Taken together, these results support the notion that introgression from local *B. rapa* accessions conferred many beneficial alleles or genes to NY7 and other Asian rapeseeds, which contributed to their shift to the semi‐winter type to adapt to Asian growth environments.

### Changes of genetic load in the NY7 pedigree accessions and Asian rapeseeds

**Figure 6:**
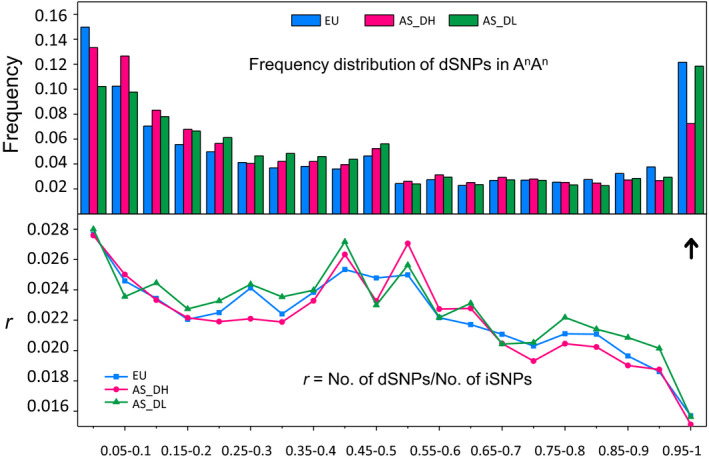
Genetic load estimation for the European and Asian groups. The top panel shows the frequency distribution of deleterious SNPs (dSNPs) in the A subgenome of European (EU) and two Asian groups (AS_DH and AS_DL) with the arrow indicating the fixed dSNPs (with a frequency of 0.95‐1 in the target population). The bottom panel illustrates the relative frequency of deleterious to neutral variants (iSNPs) in the three groups.

To better understand the dynamic changes in deleterious SNP (dSNP) frequency in Asian rapeseed populations during the different phases of breeding improvement, we estimated the dSNP allele frequency in the three rapeseed groups (EU, AS_DH and AS_DL). We found distinct dSNP frequency patterns attributable to the A and C subgenomes. Generally, the C subgenome had a much higher fixed dSNP frequency than the A subgenome in all three groups, and the most significant difference was found in the AS_DH group. The three rapeseed groups had a similar fixed dSNP frequency in the C subgenome, but AS_DH had a much lower fixed dSNP frequency than EU and AS_DL in the A subgenome (Figures 6 and S12). Consistent with this, the relative frequency of deleterious to neutral variants (intergenic SNPs) was similar among the three groups in the C subgenome but was relatively lower in AS_DH than in AS_DL and EU (Figures 6 and S13). To directly examine the effect of *B. rapa* introgression on dSNP accumulation, we further estimated the ratio between number of deleterious and intergenic SNPs (No. of dSNPs/No. of iSNPs) for each individual of the *B. napus* accessions in the NY7 pedigree. Within the pedigree, CY2 had a sudden reduction in dSNP ratio compared to its parent SL‐1 in both AA and CC subgenomes, suggesting that the lower dSNP ratio observed in CY2 could be due to the *B. rapa* accession CDA (Figure 1). We infer that the *B. rapa* hybrid event or introgression has greatly contributed to slowing the subsequent accumulation of dSNPs in Asian rapeseeds. However, NY7 had a similar dSNP ratio with SL‐1 in both the A and C subgenomes (Figure S13), possibly due to domestication costs associated with subsequent breeding improvements.

## Discussion

We generated a high‐quality Asian *B. napus* (cv. NY7) genome sequence, rigorously anchored it to an improved dense *B. napus* genetic linkage map and achieved the largest and most complete anchored genome size (892 Mb) to date. Meanwhile, we *de novo* assembled the genomes of the four progenitor accessions of NY7. These genome resources provide not only a better reference genome for *B. napus*, particularly for the Asian group but also a comprehensive genomic resource for understanding the inheritance of genetic regions underlying important traits related to adaptation of the Asian rapeseeds through inter‐ and intraspecific crosses during its breeding history. The extensive structural variations and variable deleterious SNPs observed in the two subgenomes of the European and Asian groups (Figure S5 and Table S5) enabled us to address deep questions about the processes underlying rapid genome reorganization in a young allopolyploid species.

We provided genomic evidence supporting a recent origin (<500 years) of Asian rapeseed from European rapeseed (Figure 3 and Figures S12‐13) based on the phylogenetic results of 149 global representative accessions (Malmberg *et al*., 2018). Following introduction from Europe, Asian rapeseeds have experienced strong artificial selection based on interspecific (*B. rapa*) and later intraspecific introgression events (Figures 2, 4 and 5 and Figure S16). The strong selection on traits, such as flowering time, related to local adaptation in Asian rapeseeds made them rapidly adapted to the local growing conditions. The two historical inter‐ and intra‐introgression events, combined with local selection, reshaped the genomes of the Asian rapeseeds, which highlights the high plasticity and dynamics of the polyploid rapeseed genome under artificial selection for local adaptation and targeted traits.

We observed a lower dSNP accumulation rate in the Asian double‐high group than in the European and Asian double‐low groups in the A subgenome (Figure 6), suggesting that the *B. rapa* introgression event that occurred in the 1950s slowed the accumulation of dSNPs in the Asian double‐high group. This lower rate might be due to the hybrid event that switched the derived deleterious variants of *B. napus* back to an ancestral *B. rapa* state and greatly reduced the frequency of the fixed deleterious alleles. Also, the interspecific introgression can increase the frequency of recombination events (Baurens *et al*., 2019), which may reduce the accumulation of deleterious variants. In other words, the introgression of *B. rapa* might have greatly reduced the genetic load in the Asian double‐high group and consequently enhanced their relative fitness and/or local adaptation, which may provide new insights for crop breeding.

The results achieved in this study significantly improved our understanding of the young and dynamic domesticated genome of *B. napus*, particularly the differentiation and local adaptation of Asian and European rapeseeds. It is apparent that both the interspecific introgression from the *B. rapa* A genome and the subsequent intraspecific crossing between the Asian and European *B. napus* groups have significantly broadened the gene pool of modern *B. napus*. It appears that the strategy to reintroduce the traits from the diploid progenitor with a better local adaptation has contributed to decreasing genetic load towards a rapid adaptation in new environments in a short period of time.

### URLs

BnPedigome ( http://ibi.zju.edu.cn/bnpedigome/), a genomic database for the pedigree and breeding history of *B. napus* cultivars*,* developed by this study; BRAD, http://brassicadb.org/brad/; GENOSCOPE, www.genoscope.cns.fr/brassicanapus/.

## Methods

### Plant materials and phenotypes

Ningyou7 (NY7), a Chinese semi‐winter cultivar with double‐high seed quality (high glucosinolates and high erucic acid), its four parents in the pedigree (SL‐1: Shengliyoucai‐1; CDA: Chengduaiyoucai; CY2: Chuanyou2; and NY1: Ningyou1) and Zhongshuang11 (ZS11, a double‐low cultivar) were analysed in this study (Figure 1a). There are several rapeseed accessions with the same name as ‘Shengliyoucai’ (SL; *B. napus*), including the original accession imported from Japan and a few Chinese cultivars derived from the imported SL in different breeding programmes. To determine the founder parent SL in the NY7 breeding pedigree, four SL accessions (three provided by the Germplasm Research Group of the Oilseeds Research Institute, Chinese Academy of Agricultural Sciences) were collected and sequenced to at least 15 × coverage (Table S1). A phylogenetic tree of *B. napus* accessions including the four SL accessions was constructed based on genome‐wide SNPs (Figure S7). The SL accession closest to NY7 was named SL‐1 and considered to be the parental line of NY7. This SL accession was further deep‐sequenced and used in subsequent genome assembly and sequence analysis (Table 1). Meanwhile, publicly available genomic data of 160 *B. napus*, 34 *B. rapa* and 37 *B. oleracea* accessions were also used in this study (Table S6). The 160 *B. napus* accessions include 59 European, 38 Asian double‐high and 30 Asian double‐low rapeseeds.

### Mapping population and genetic map construction

A double haploid (DH) population (*Bna*TNDH) with 353 DH lines derived from crossing NY7 with a European winter cultivar Tapidor was used to construct the genetic map. This population has been used as a reference genetic mapping population internationally and has accumulated much genotypic and phenotypic data (Qiu *et al*., 2006; Zhang *et al*., 2016). The 353 DH lines were genotyped using the Illumina Infinium *Brassica* 60K SNP array. The SNP markers were filtered with high‐quality parameters, less than 0.05% missing data and MAF ≥ 0.1. The markers showing the same segregation pattern were classified into the same genetic bin. For each genetic bin, the SNP marker with the highest quality was chosen to construct the genetic map using JoinMap 4.0 (Van Ooijen, 2006). The final genetic map (*Bna*TNDH 2.3) contains 2964 genetic bins, including 14 936 SNP markers (Table S2). Double crossovers and recombination frequency were checked by Mapdisto 2.0 (Lorieux, 2012).

### Genome sequencing, assembly and annotation

#### Genome sequencing

NY7 genome sequencing was performed through a combination of sequencing technologies, including Illumina, PacBio Single Molecule Real Time (SMRT) and Hi‐C sequencing (see Table S1 for the detailed information of sequencing data). For Illumina sequencing, 160 bp‐20 Kb insertion libraries were constructed (Table S1); the Hi‐C experiments and sequencing procedures were similar to those described previously in cotton (Wang *et al*., 2017a). The four parental lines in the NY7 breeding pedigree (NY1, CY2, SL‐1 and CDA) and European lines Tapidor were deep‐sequenced with Illumina technology with 300 bp‐15 Kb insertion libraries. The other three SL accessions (SL‐2, SL‐3 and SL‐4) were also resequenced to over 15 × coverage using Illumina technology (Table S1).

#### *De novo* assembly of scaffolds

To avoid systematic bias from sequencing reads, the raw Illumina paired‐end (PE) reads were filtered using NGSQC v2.3.3 (Patel and Jain, 2012) and corrected using Lighter (Song *et al*., 2014) with the default setting. The clean Illumina reads were assembled using SOAPdenovo2 (Luo *et al*., 2012). Further, the clean reads were reused to improve the SOAPdenovo2 assembly using Gapcloser v2.1 (Luo *et al*., 2012), SSPACE v1.0 (Boetzer *et al*., 2011) and OPERA‐LG v2.1 (Gao *et al*., 2016). Finally, the PacBio reads corrected by the clean Illumina reads with the software LoRDEC 0.6 (Salmela and Rivals, 2014) were used to fill the gaps by PBJelly (English *et al*., 2012). To reduce errors in the initial assembly, three linkage maps and the A^r^ and C^o^ subgenomes were applied to identify and correct chimeric scaffolds according to the dependable synteny relationship with the initial NY7 assembly. As a result, a total of 180 chimeric scaffolds were corrected, and the NY7 assembly was improved to have a scaffold N50 1.27 Mb and a contig N50 44.0 Kb.

#### Super‐scaffolding with Hi‐C data

Approximately, a total of 117 Gb Hi‐C reads were mapped to the assembly using Bowtie v2.2.1 (Langmead and Salzberg, 2012) with parameters ‘–reorder’ and ‘–very‐sensitive’. The software SAMtools (Li *et al*., 2009) was used to manipulate the BAM files and remove potential PCR duplicates. Then, we used Lachesis (Burton *et al*., 2013) to cluster, order and orientate the scaffolds and created the raw Hi‐C assembly with the mapping result in the last step. For accuracy of the assembly, the Hi‐C contact matrix was generated, and custom scripts were used to find and split these weak points among the Hi‐C assembly. The corrected Hi‐C assembly was then aligned to the three linkage maps and the A^r^ and C^o^ subgenomes to remove the abnormal synteny relationship. Finally, we obtained the NY7 Hi‐C assembly with a scaffold N50 6.91 Mb.

#### Pseudo‐molecule construction

The backbone linkage map, *Bna*TNDH 2.3 (Table S2), and two other published linkage maps, DYDBAA (Chalhoub *et al*., 2014) and BS (Delourme *et al*., 2013), were used to construct the pseudo‐molecules. A final set of 13 164 unique SNP markers was utilized to anchor the Hi‐C scaffolds using blast +2.3.0 with the parameter ‘‐evalue 1e‐10’. The markers with the best hits in the NY7 genome, and further filtrated using Allmaps (Tang *et al*., 2015), were defined as unique markers. Allmaps was used to construct the 19 pseudo‐chromosomes that covered 890 Mb in length.

#### Improving the assembly of NY7 with GOGGs

Illumina RNA‐seq reads from 45 DH lines of the *Bna*TNDH mapping population and 2 parental lines (NY7 and Tapidor) were mapped to the NY7 reference genome using the methodology developed and deployed previously (Bancroft *et al*., 2011). Genome‐ordered graphical genotypes (GOGGs) by He and Bancroft (2018) were applied to improve the genome assembly of NY7. In brief, we developed a new NY7 genome sequence resource by cutting and inserting 54 segments from the draft genome sequence based on GOGGs (Table S3‐1), and the reassembly of the split segments was designed based on the congruence of genotypes of the mapping population. The new NY7 genome sequence was reassembled based on an automation of concatenating the sequence segments. Another iteration of SNP scoring and generation of GOGGs from this resource demonstrated the improved congruence of genotypes (Table S3‐2).

#### Assembly confirmation with Hi‐C contact maps

~100x Hi‐C data were remapped to the 19 pseudo‐chromosomes and normalized using HiC‐Pro 2.10.0 (Servant *et al*., 2015) with the parameters ‘FILTER_LOW_COUNT_PERC = 0 and BIN = 100000’. The Hi‐C contact maps with a bin of 100 Kb generated by HiC‐Pro were used to plot Hi‐C contact heatmaps for the 19 pseudo‐chromosomes using HiCPlotter 0.7.3 (Akdemir and Chin, 2015) with parameters ‘‐tri 1 ‐wg 1 ‐o WholeGenome’ (Figure S2).

#### Gene and repeat annotation

We built the *de novo* repeat library from the assembled genome using RepeatModeler (Chen, 2009). A total of 352.8 Mb repetitive elements covering 41.29% of the NY7 genome were identified using RepeatMasker 4.0.8 (Chen, 2009) with the default settings. *De novo* gene structure predictions were carried out using AUGUSTUS 3.2.2 (Stanke *et al*., 2006), GeneMark.hmm (Lukashin and Borodovsky, 1998) and FGENESH 2.6 (Salamov and Solovyev, 2000). The coding sequences of *A. thaliana* (TAIR10), *B. rapa* (IVFCAASv1), *B. napus* (Darmor‐*bzh,* v5 and ZS11 (V201608)) and *B. oleracea* (v2.1) were downloaded to perform the homology predictions using gmap (Wu and Watanabe, 2005). A total of ~20 Gb RNA‐seq reads generated by Bancroft *et al*. (2011) were aligned to the NY7 assembly using Tophat 2.1.1 (Trapnell *et al*., 2009) and assembled to a set of transcripts using Cufflinks 2.2.1 (Trapnell *et al*., 2010) with the default settings. Meanwhile, the RNA‐seq data were assembled using Trinity 2.4.0 (Grabherr *et al*., 2011) with the default settings. The assembled transcripts from Cufflinks and Trinity were integrated using the PASA 2.0.2 (Haas *et al*., 2003) pipeline to provide expression evidence for gene predictions. All candidate gene models from the above evidence were combined using EVM (Haas *et al*., 2008) with a higher weight for the expression evidence from RNA‐seq results.

### Detection of genomic variations

Clean reads of each accession were mapped to the newly assembled NY7 reference genome and the Darmor‐*bzh* genome using Bowtie v2.2.1 (Langmead and Salzberg, 2012) with the default settings. SAMtools v0.1.19 (Li *et al*., 2009) and GATK v2.3 (McKenna *et al*., 2010) were applied for variant detection using parameters similar to those described previously (Qiu *et al*., 2017). The SNP calls were filtered according to the following threshold: QUAL < 30, DP < 10, QD < 2. Potential variant annotation and effect were predicted by SnpEff v3.6 (Cingolani *et al*., 2012). The structural variations between NY7, Darmor‐*bzh* and other genomes were detected by SVMU (Chakraborty *et al*., 2018). Each NY7 chromosome was aligned to the corresponding chromosome of Darmor‐*bzh* by MUMmer (nucmer –mumreference –noextend).

### Identity‐by‐descent (IBD) blocks

To determine the IBD origin of NY7 genomic segments, we took advantage of our *de novo* assembled genomes of the four parental lines of NY7. The NY7 genome was first divided into blocks with a length of 50 Kb. All scaffolds of the four parental lines were taken as a reference genome database for tracing the sequence origin of NY7. MUMmer3.23 (Kurtz *et al*., 2004) was applied to perform the 1‐to‐1 alignment. We calculated the match length between NY7 and its parental lines in each 50 Kb block and assigned the origin of the block to the line that has the longest accumulated alignment length. The same pipeline of IBD analysis was applied to trace the sequence origin of CY2 from SL‐1 and CDA. The demonstration of IBD in each chromosome was drawn with the Perl SVG module.

### Phylogenetic and PCA

SNPs were filtered with VCFtools with parameters ‘‐maf 0.01 ‐max‐missing 0.9’ for the *B. napus* accessions used in this study (a total of 127 lines, see Table S7). A phylogenetic tree was constructed using Fasttree (Price *et al*., 2009) with 1000 replicates for bootstrap confidence analysis, and MEGA v7 (Kumar *et al*., 2016) was used to draw the tree. Principal component analysis (PCA) was performed by SNPRelate v0.9.19 (Zheng *et al*., 2012).

### Demographic analysis

SNPs in intergenic regions were used to minimize bias in demographic analyses due to selection. The best parameters for fitting the model were estimated by ∂a∂i v1.6.3 (Gutenkunst *et al*., 2009). The alleles were down‐sampled via hypergeometric projection for each group. The folded spectrum was used for each pool. Four demographic models were considered for each type (Table S7 and Figure S9). For inference of Asian double‐low rapeseed, five demographic models were considered (Table S7 and Figure S10). Different demographic models were compared with the basis of the relative log likelihoods of the models, given the observed site frequency spectrum. Thirty independent runs with randomized starting points were executed for each candidate model, and the average value was chosen based on the best fitting parameters.

### Selection analysis

The genome was scanned in a 100 Kb window size, and the population parameters (π, *F*_ST_) were estimated for each window by VCFtools (Danecek *et al*., 2011). Nucleotide diversity (π) was measured with parameters ‘–window‐pi 100 000 –window‐pi‐step 10 000’. For measurement of population differentiation, *F*_ST_ was calculated with the setting ‘–fst‐window‐size 100 000 –fst‐window‐step 10 000’. *Z*‐transformation was applied to locate divergent regions from the extreme tails by applying a threshold of 3 standard deviations. The nonredundant genes residing in these regions were taken as putatively divergent genes between different populations.

### Locating QTL to the NY7 assembly

QTL for seed yield reported by Luo *et al*. (2017) were mapped to the NY7 assembly using BLAST+2.3.0 with parameters ‘‐evalue 1e‐10’. After removing the markers locating different pseudo‐chromosomes with linkage maps or having abnormal physical location with adjacent markers in the linkage map, 303 TLs, each with at least two unique markers, were mapped in the NY7 assembly.

### Local ancestry inference

Local ancestry inference was performed using Loter (Dias‐Alves *et al*., 2017), which can use haplotype data to infer ancestry across the chromosomes of an admixed individual from two proposed ancestor populations. For inference of potential *B. rapa* introgression in Asian rapeseed, twelve Asian rapeseeds for oil use and European rapeseed were employed as parental populations. For inference of potential European lineages in Asian double‐low cultivars, European rapeseed and Asian double‐high rapeseed populations were employed as the two parental populations.

### Identification of deleterious mutations

Deleterious SNPs (dSNPs) were predicted using SIFT (Kumar *et al*., 2009). The combined *B. rapa* and *B. oleracea* genomes (EnsemblePlants, release‐37) were used for building the reference database, considering that using either the Asian (NY7) or European (Darmor‐*bzh*) rapeseed genome as the reference would substantially cause reference bias for dSNP identification. All genomic resequencing data of *B. napus* individuals were mapped to the combined reference for SNP calling and genotyping. A dSNP was defined if the value calculated by SIFT for a SNP had a normalized probability <0.05. To calculate the site frequency spectra for Asian and European populations, the number of deleterious alleles in a population was calculated as twice the number of homozygous variants plus the number of heterozygous variants.

## Author contributions

L.F. and J.M. conceived and designed the project. J.Z. and W.M. collected materials, performed the experiments, developed genetic maps, and performed QTL analysis and result analysis. L.M. and J.Q. performed genome assembly. J.Q., L.M., L.J., D.W., N.S., Y.S., J.Z., M.W., R.L., D. H., M.C., C.Y. and L.F. managed sequencing and analysed the data. Z.H. and I.B. performed GOGGs for genome assembly corrections. J.M., I.B., L.S. and C.Y. discussed the data. J.Q., J.Z., L.M. and L.F. wrote the manuscript. Q‐H. Z., G.K., I.B. and J.M. revised the manuscript.

## Competing financial interests

The authors declare no competing financial interests.

## Supporting information


**Figure S1** Genomic survey of NY7 and its pedigree accessions with *K*‐mer distribution.
**Figure S2** The Hi‐C contact maps for the whole genome (left panel) and chrA03 (right panel) of NY7.
**Figure S3** The collinearity between each of the three *B. napus* genetic maps and the NY7 genome assembly.
**Figure S4** The pipeline for genome assembly and pseudo‐chromosome construction of NY7.
**Figure S5** The genomic synteny between NY7 and other *de novo* assemblies of *B. napus* and two diploid progenitors. a‐e present the synteny between NY7 and Darmor‐*bzh*, progenitor diploid *B. rapa* and *B. oleracea*, Tapidor reported by Bayer *et al*., 2017, Tapidor updated in this study, and ZS11, respectively; f presents the synteny between the two Tapidor assemblies, the one generated in this study and the one reported by Bayer *et al*., 2017.
**Figure S6** Demonstration of the QTL located in the genomic divergent region between the NY7 and Tapidor genomes. Genomic alignment view of chromosome C03 of the Tapidor assembly mapped to the NY7 reference. All QTL identified in C03 were labelled with ‘es.’ at the top of the rectangle. The arrow shows a QTL (*es. C3‐3*) region with present and absent sequence variation between Tapidor and NY7. Significantly high read coverage in the NY7 genome was found in the QTL (*es. C3‐3*) region.
**Figure S7** Phylogenetic trees for *B. napus* accessions based on SNPs found in both the A and C subgenomes. The EU, AS_DL, AS_DH represent the European rapeseed (*B. napus*), Asian rapeseed (*B. napus*) with double‐low and double‐high traits, respectively.
**Figure S8** Genetic diversity (π) of different chromosomes in different populations. EU and AS represent the European rapeseed (*B. napus*) and Asian rapeseed (*B. napus*), respectively. The EU, AS_DL, AS_DH represent European rapeseed, Asian rapeseed with double‐low and double‐high seed‐quality traits, respectively.
**Figure S9** Linkage disequilibrium of the European and Asian rapeseed populations.
**Figure S10** Demographic models used in assessing the origin of Asian rapeseeds.
**Figure S11** Demographic models for assessing the origin of Asian double‐low rapeseeds.
**Figure S12** A genomic view of the reads mapped to the *CDF2* homologue in the parental lines of NY7.
**Figure S13** The frequency distribution of deleterious SNPs (dSNPs) and neutral intergenic SNPs (iSNPs) in (a) the NY7 pedigree accessions (b) the C^n^ genome of the Asian and European populations.
**Figure S14** (a) Local ancestry inference of the introgression of *B. rapa* for Asian rapeseed. On the top of each box, the red signals represent the conserved blocks with introgression from *B. rapa*. (b) Local ancestry inference of the introgression of European lineage (blue) in Asian double‐low cultivars.
**Figure S15** (a) A genomic view of NY1 reads in the homoeologous chromosome pair A01 and C01 with two diploid ancestors (*B. rapa* and *B. oleracea*) as references. (b) A genomic view of NY1 and CY2 reads in the homoeologous chromosome pair A09 and C09 with two diploid ancestors (*B. rapa* and *B. oleracea*) as references.
**Figure S16** A summary of the origin and breeding history of Asian rapeseeds and the dynamic changes of genomic diversity and deleterious SNP (dSNP) accumulations in the two stages of the Asian local breeding history. The Asian genomes were significantly re‐shaped by two genetic introgression events contributed by *B. rapa* and double‐low European *B. napus*.

**Table S1** Summary of the sequenced genomic data in this study.
**Table S2** The linkage genetic map of the *Bna*TNDH mapping population (version *Bna*TNDH 2.3).
**Table S3** Re‐assembly of the NY7 genome according to the genome‐ordered graphical genotypes constructed using the *Bna*TNDH population.
**Table S4** Genome completeness assessment using CEGMA and BUSCO.
**Table S5** Number of different types of genomic variations between NY7 and other *de novo* assembly genomes.
**Table S6** *Brassica* accessions used in this study.
**Table S7** Details of the demographic model for assessing the origin of Asian rapeseeds.
**Table S8** Divergent regions and associated QTL between the Asian and European cultivars.
**Table S9** The conserved regions introgressed from *B. rapa* in the Asian double‐high rapeseeds and QTL located in these regions.
**Table S10** QTL in the IBD genomic blocks derived from CDA.
**Table S11** Nonsynonymous SNPs found in the genes related to flowering time and their frequency in the Asian and European populations.

## Data Availability

All the sequence data sets generated during the current study are available in the NCBI BioProject under accession PRJNA526961. All genome assemblies and annotations can be downloaded in http://ibi.zju.edu.cn/bnpedigome/download.php.
