# Supplementary material for: Genome‐wide selection footprints and deleterious variations in young Asian allotetraploid rapeseed
Source: Plant Biotechnol J. 2019 Apr 17;17(10):1998–2010. doi: 10.1111/pbi.13115 (PMC6737024; doi:10.1111/pbi.13115)

Figure S1 Genomic survey of NY7 and its pedigrees with *K*-mer distribution

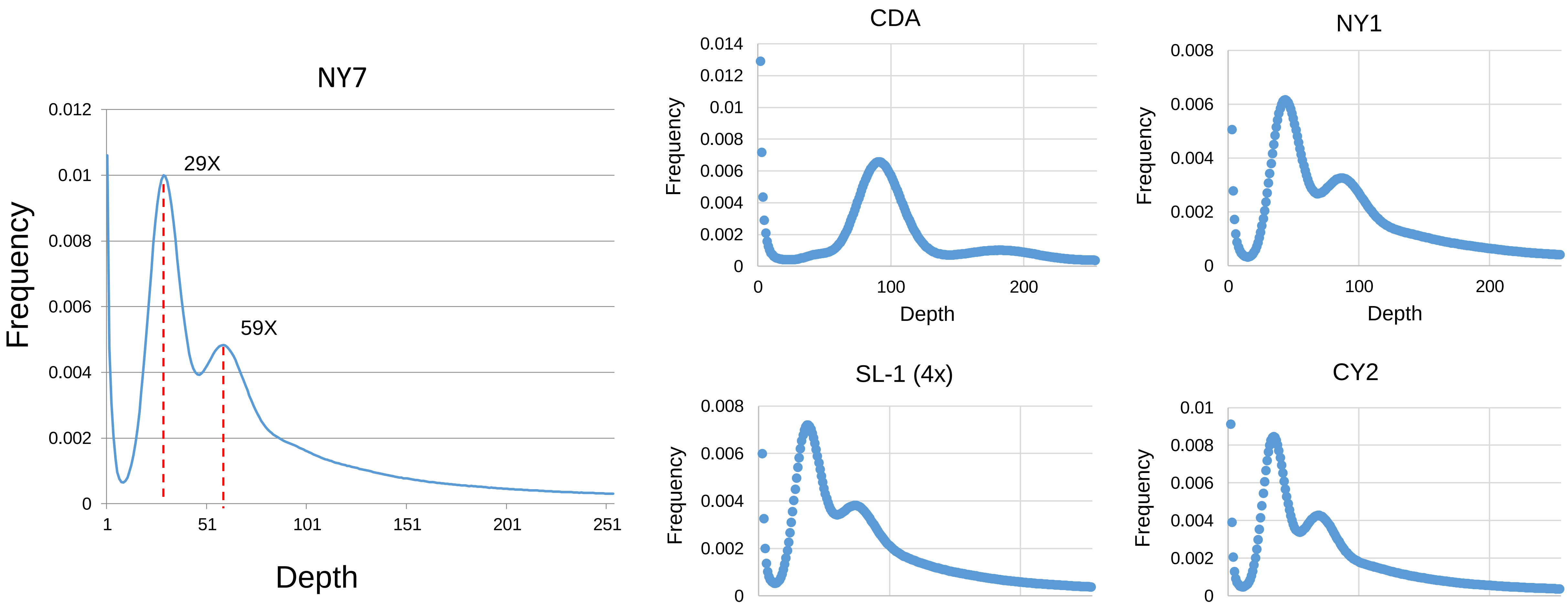

Figure S2 The Hi-C contact maps for NY7 whole genome and NY7 chrA03

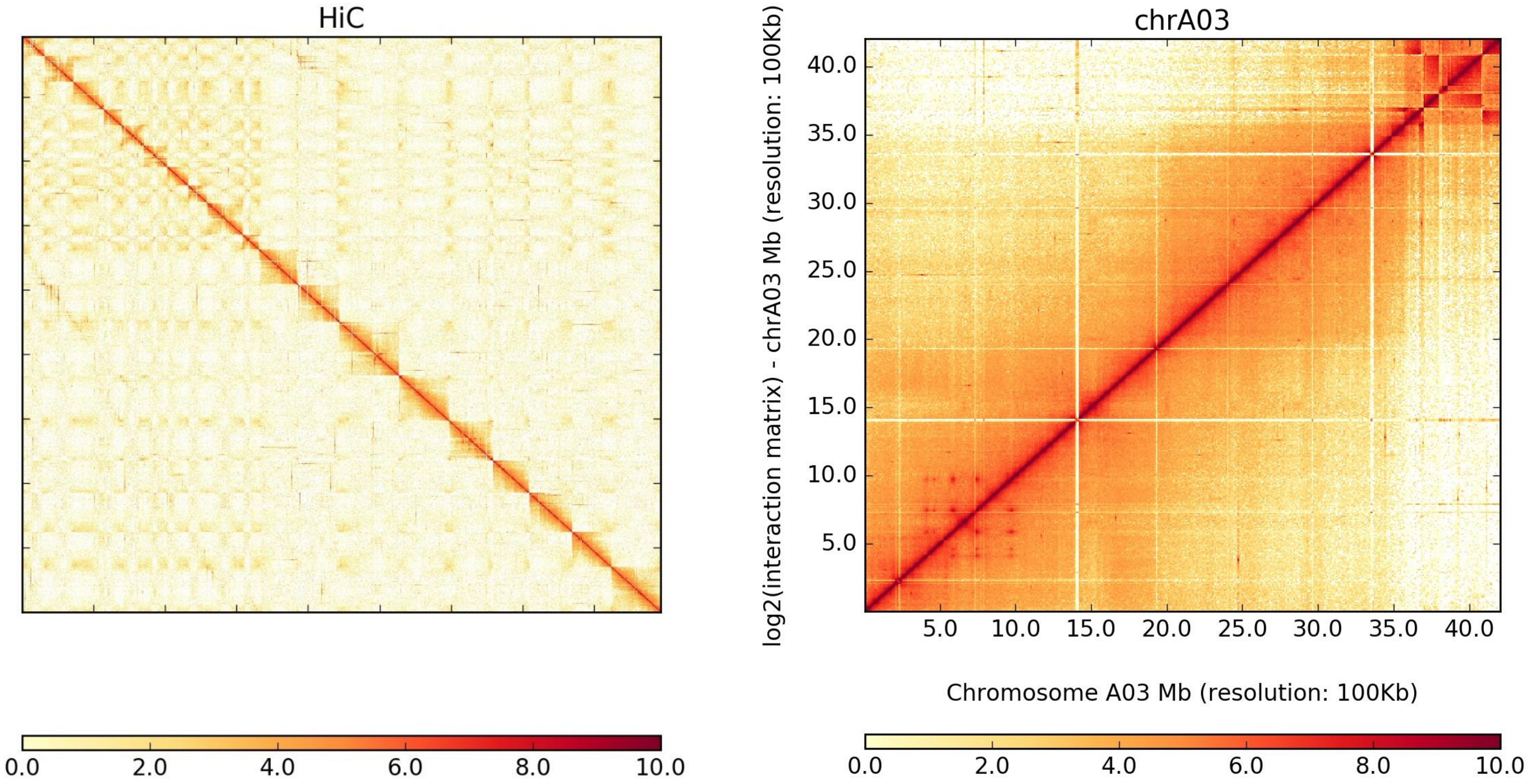

Figure S3 The collinearity between three *B. napus* genetic maps and the NY7 genome assembly

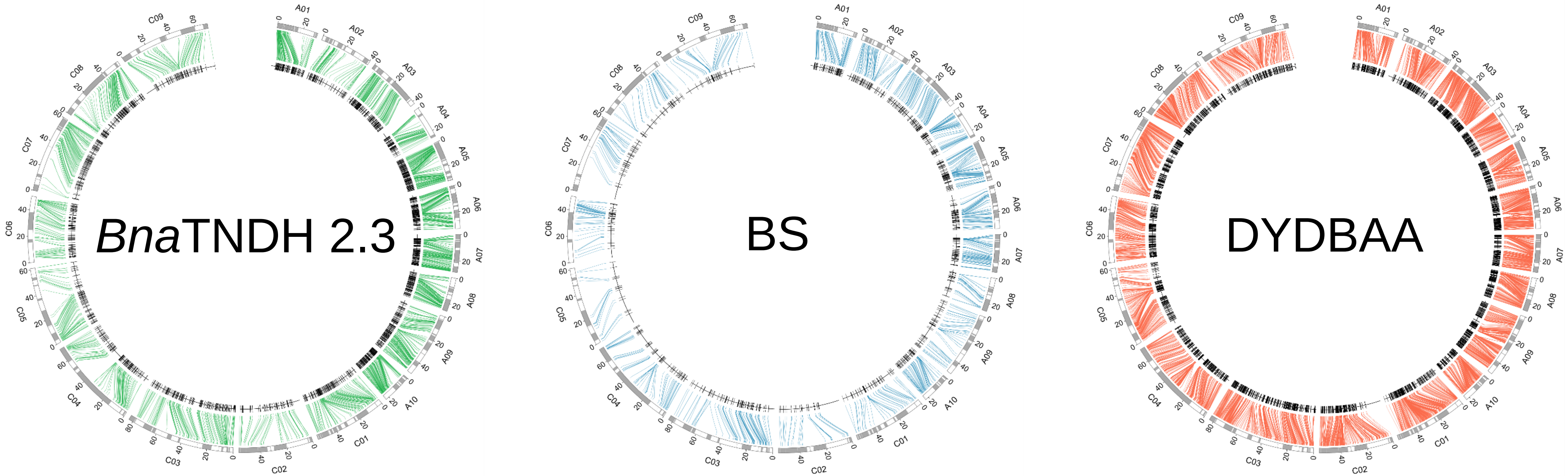

Figure S4 The pipeline for genome assembly and pseudo-chromosome construction for NY7

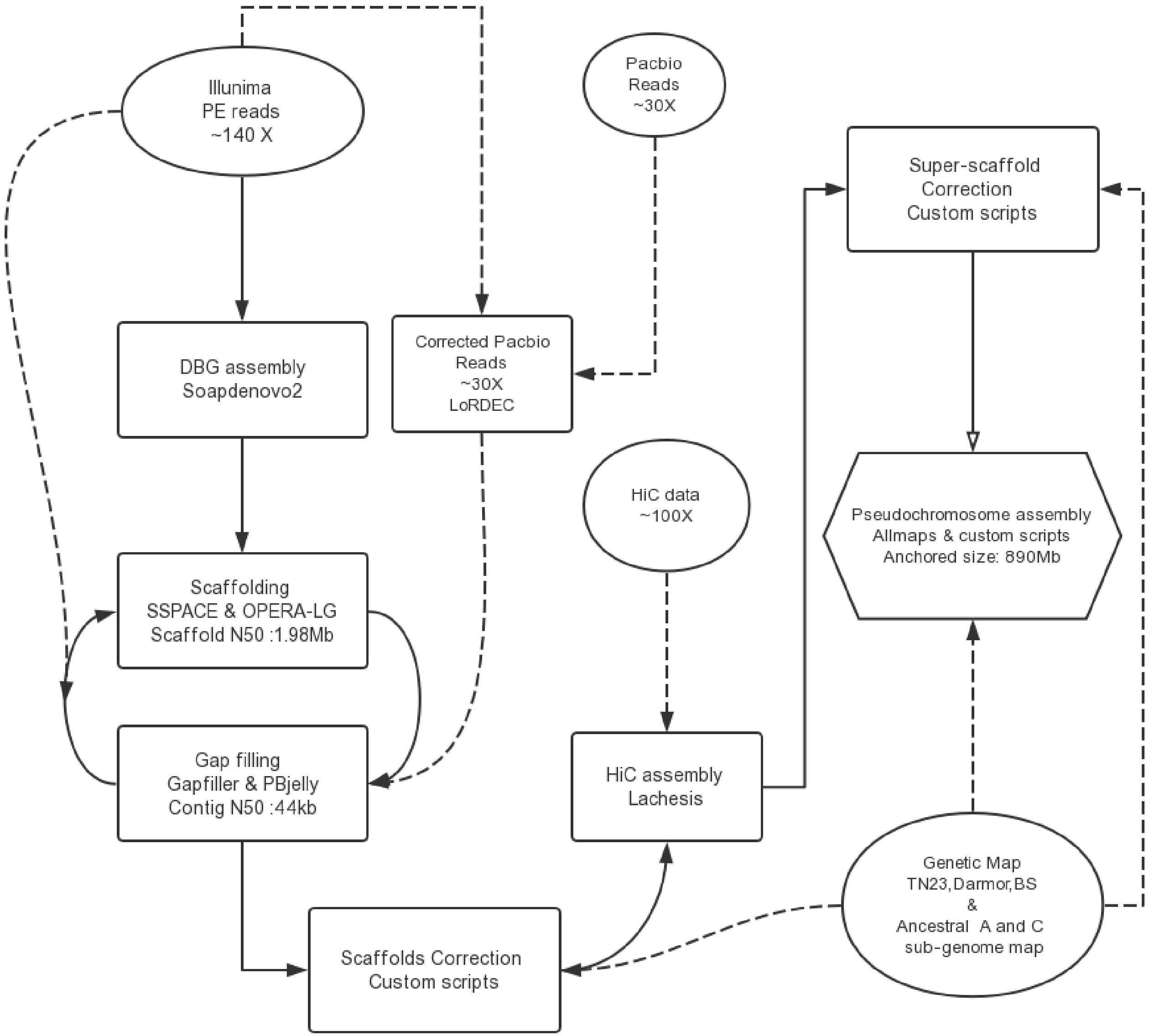

Figure S5 The genomic synteny between NY7 and other *de novo* assembly of *B. napus* and two diploid progenitors

a

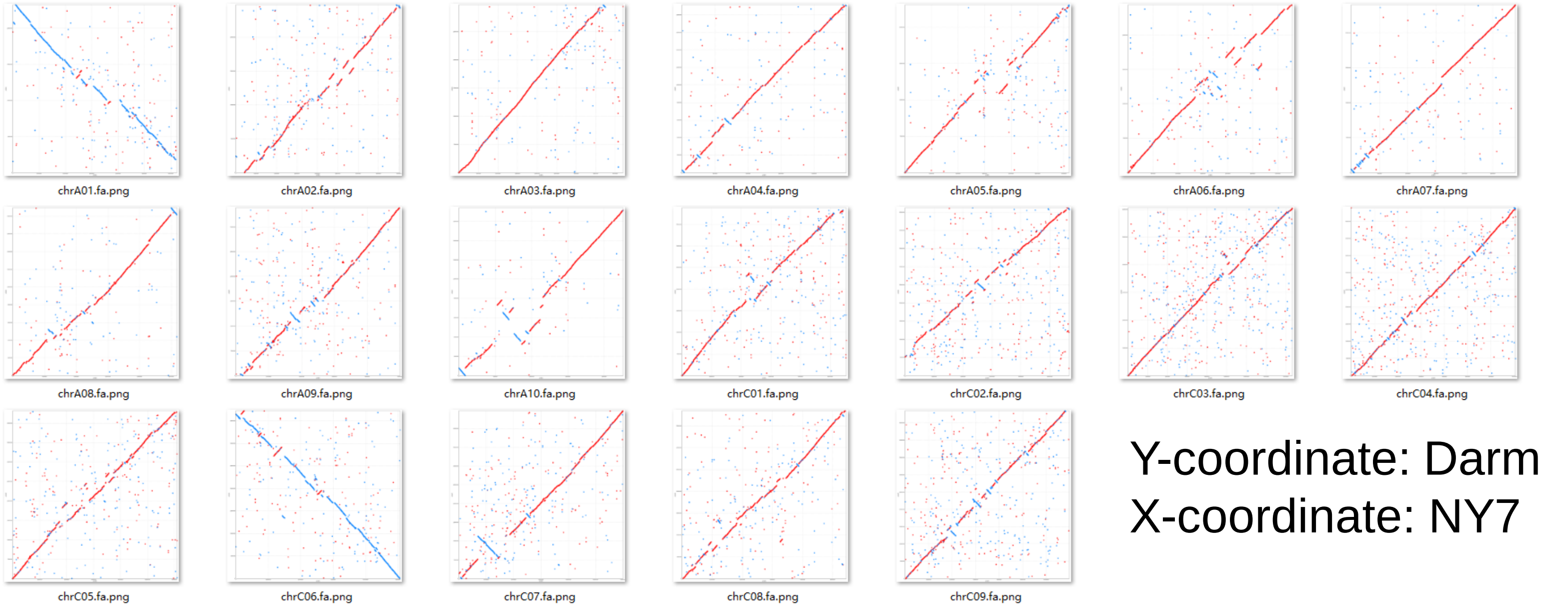

Y-coordinate: Darmor-*bzh*
X-coordinate: NY7

b

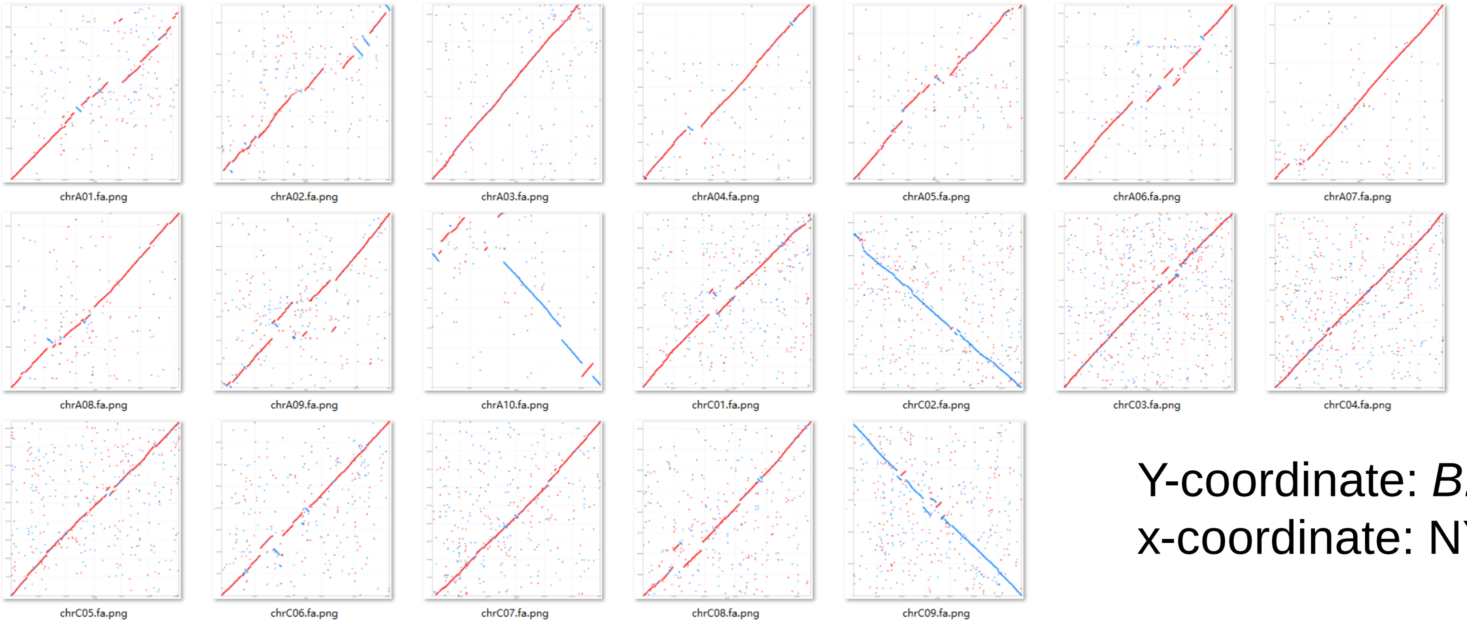

Y-coordinate: *B. rapa* and *B. oleracea*
x-coordinate: NY7

Figure S5 The genomic synteny between NY7 and other *de novo* assemblies of *B. napus* and two diploid progenitors

c

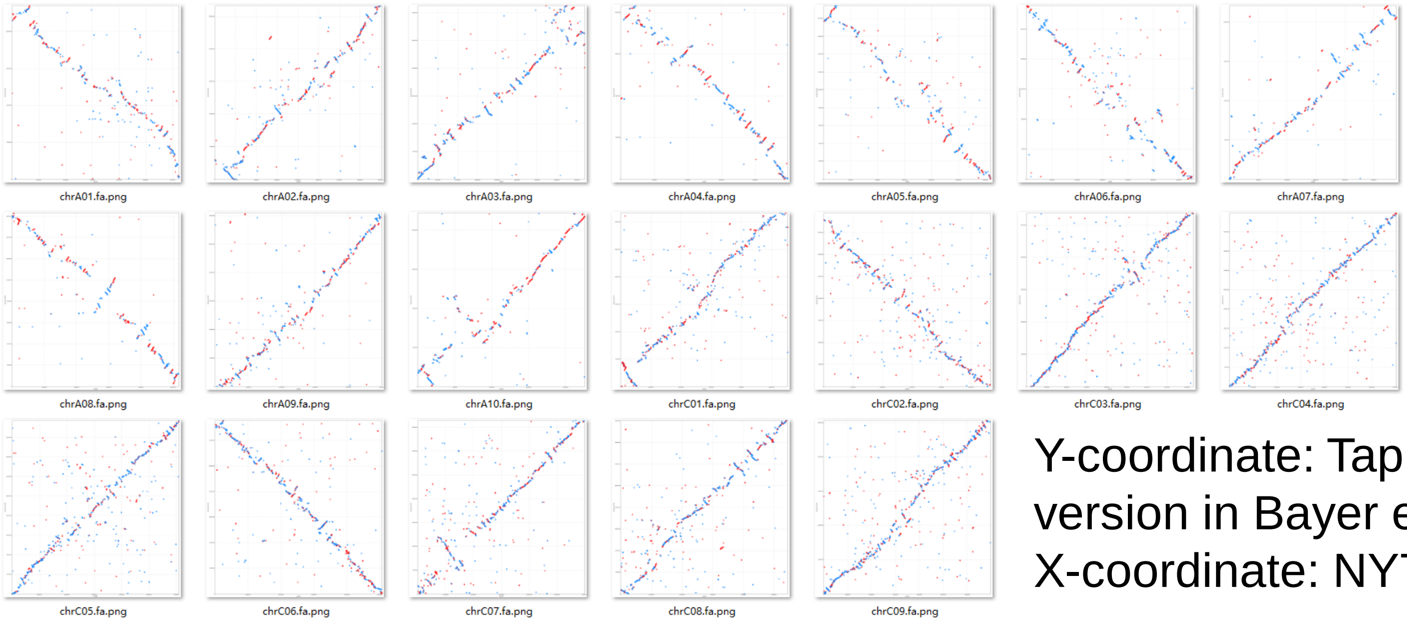

Y-coordinate: Tapidor_published version in Bayer et al. 2017
X-coordinate: NY7

d

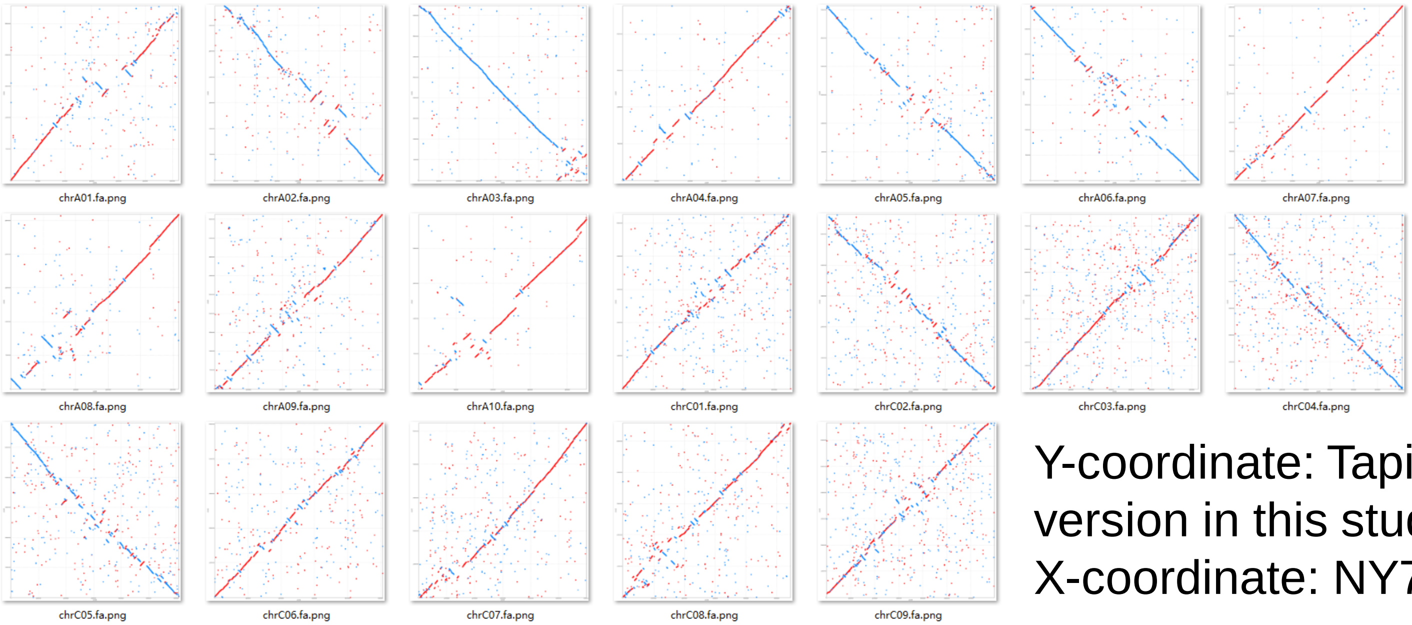

Y-coordinate: Tapidor_updated version in this study
X-coordinate: NY7

Figure S5 The genomic synteny between NY7 and other *de novo* assemblies of *B. napus* and two diploid progenitors

e

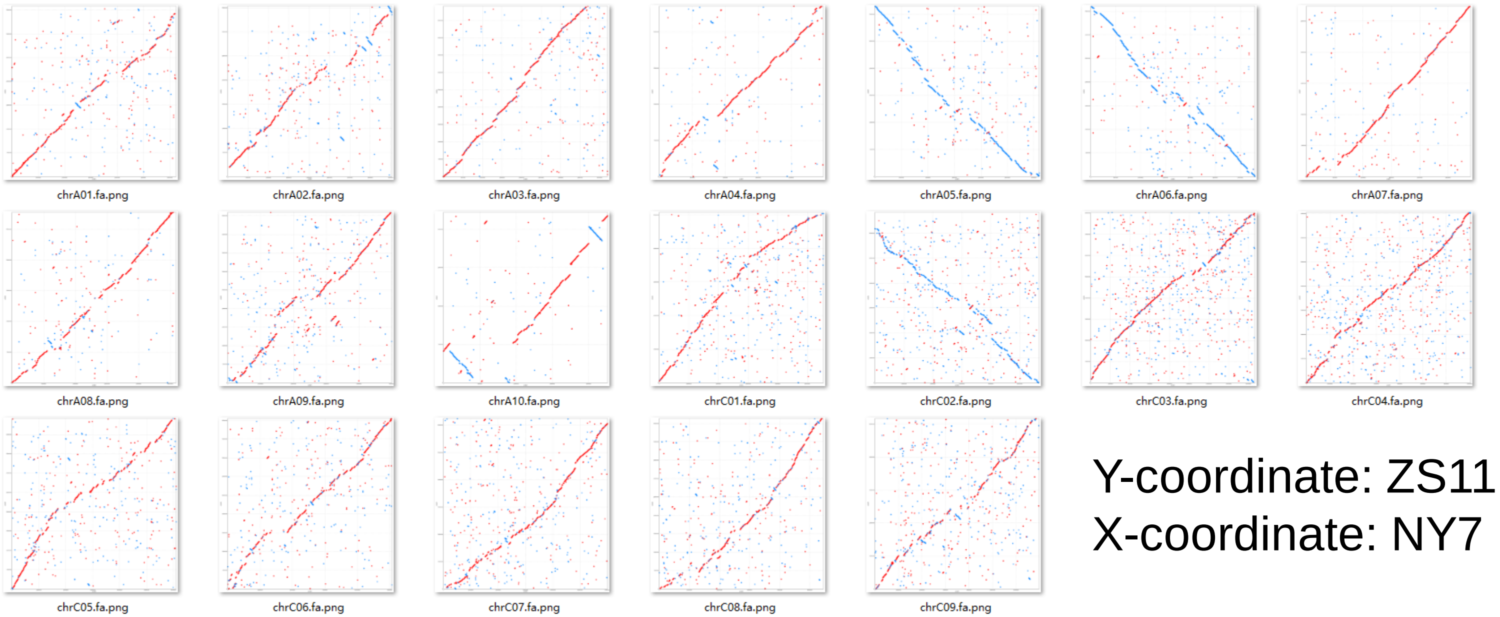

Y-coordinate: ZS11
X-coordinate: NY7

f

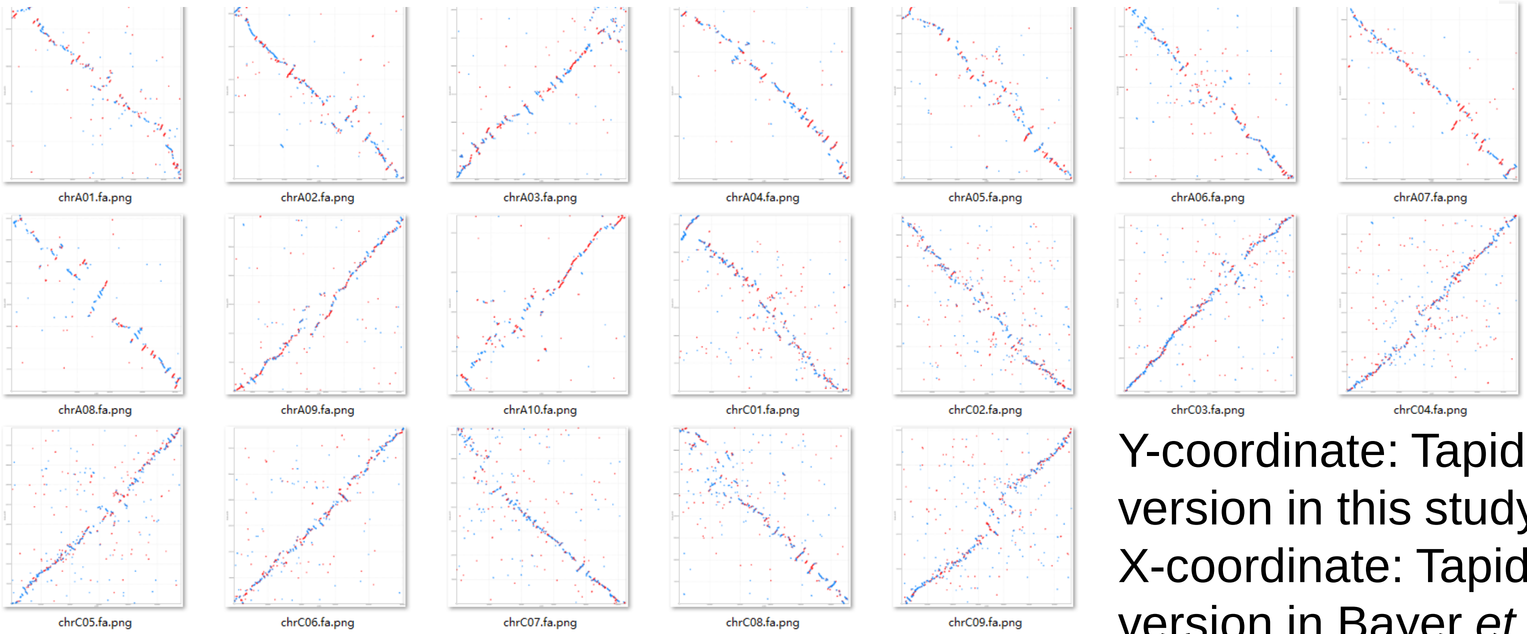

Y-coordinate: Tapidor_updated version in this study
X-coordinate: Tapidor_published version in Bayer *et al*. 2017

Figure S6 Demonstration of a QTL located at the genomic divergent region between the NY7 and Tapidor genome

Genomic alignment view of the Tapidor assembly to NY7 reference on chromosome C03. All of the QTLs identified in C03 were labelled with ‘es.’at the top. The arrow shows a QTL (*es. C3-3*) region with present and absent sequence variation between Tapidor and NY7. Significantly high read coverage in the NY7 genome was found in the QTL (es. C3-3) region.

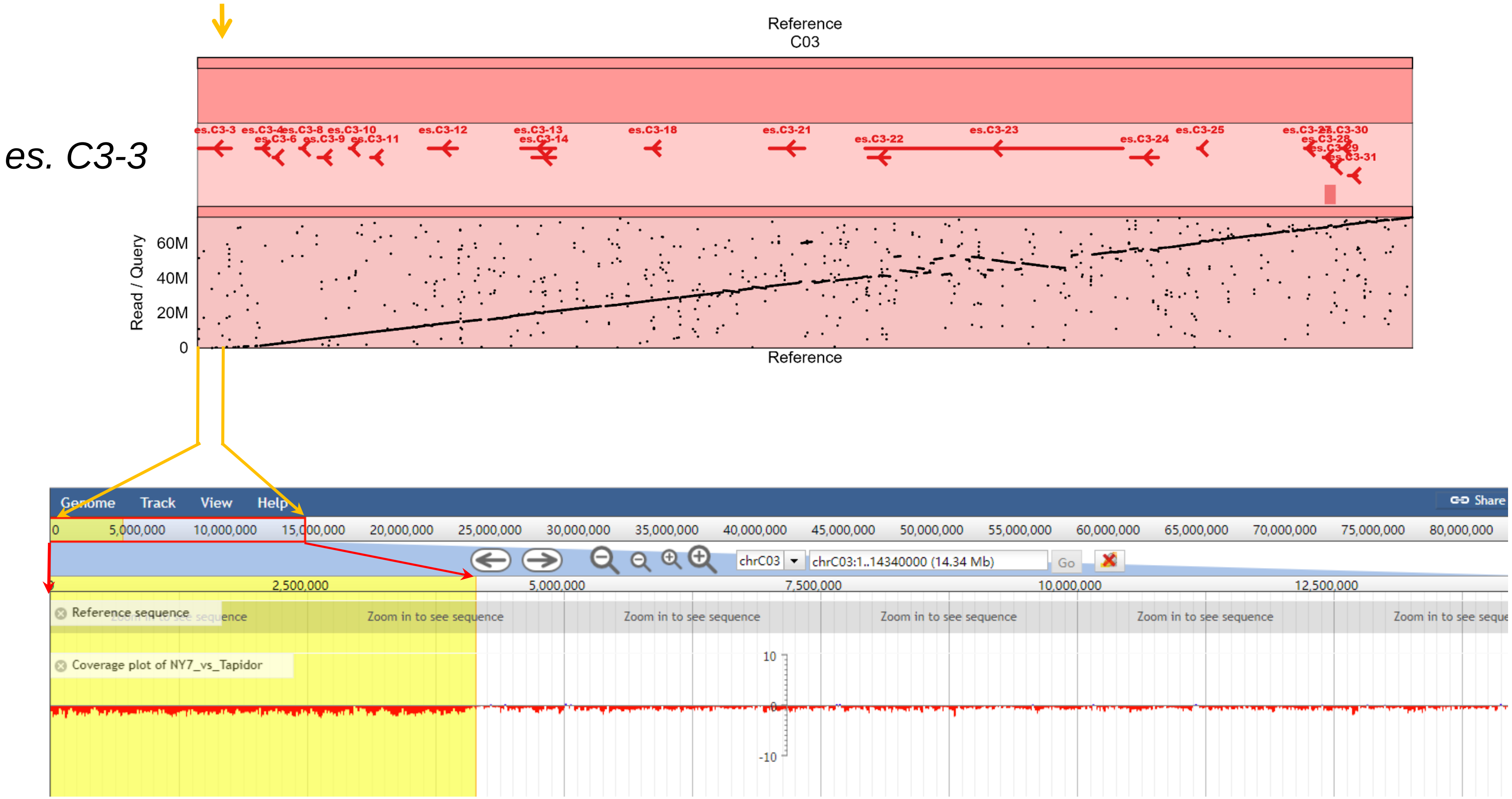

Figure S7 Phylogenetic trees for *B. napus* accessions based on SNPs found in both the A and C subgenomes

The EU, AS_DL, AS_DH represent the European rapeseed (*B. napus*), Asian rapeseed (*B. napus*) with double-low and double-high traits, respectively.

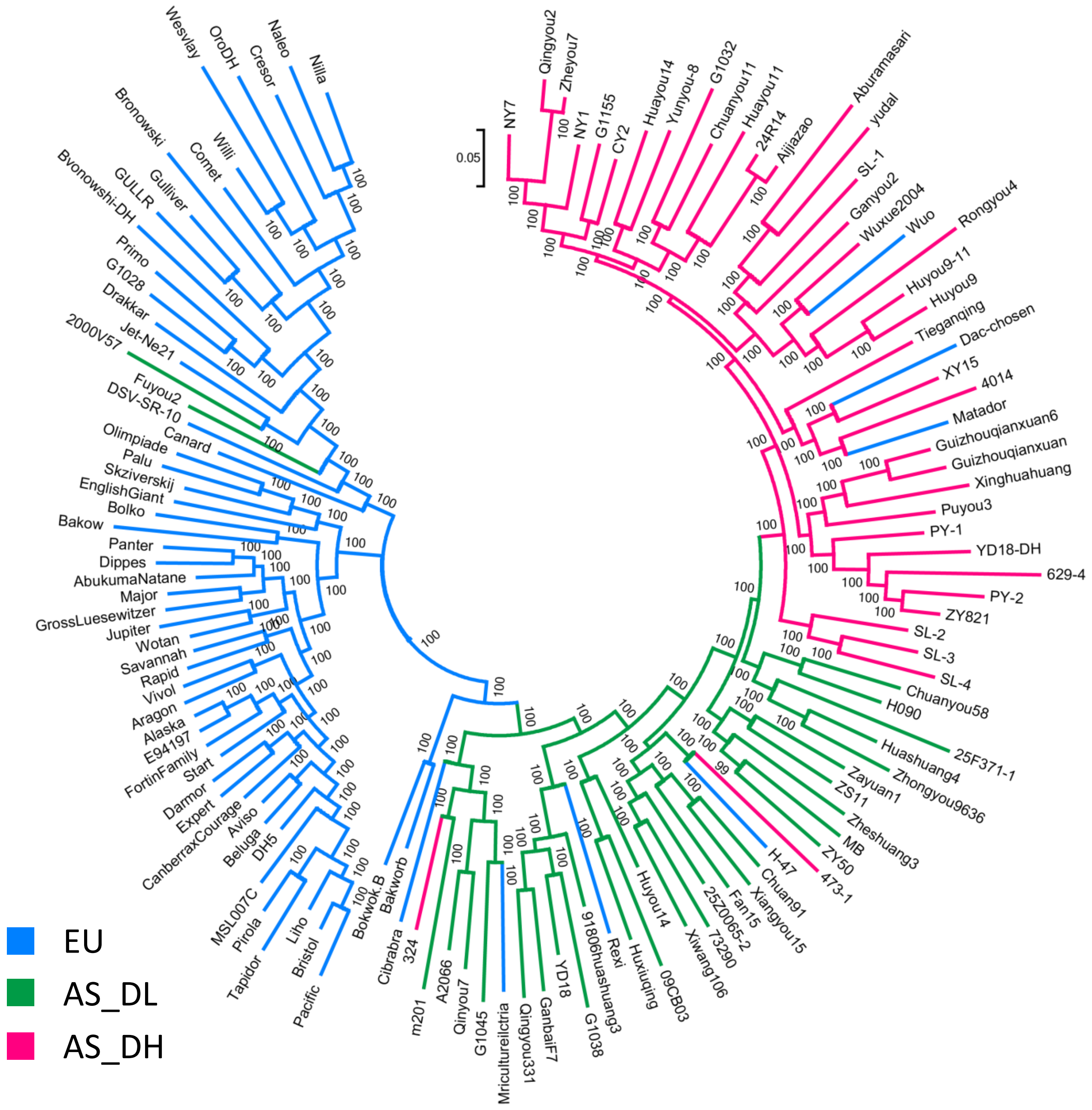

Figure S8 Genetic diversity (π) of different chromosomes in different populations. EU and AS represent the European rapeseed (*B. napus*) and Asian rapeseed (*B. napus*), respectively. The EU, AS_DL, AS_DH represent European rapeseed, Asian rapeseed with double-low and double-high seed-quality traits, respectively

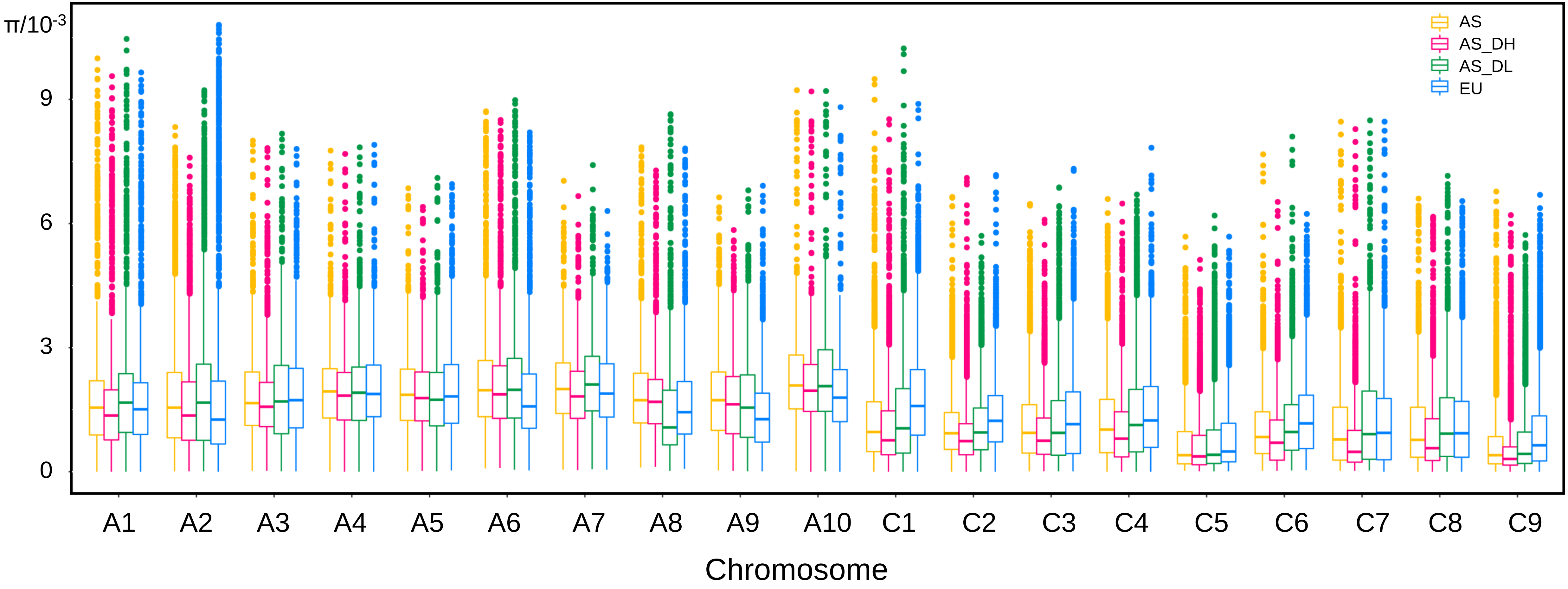

Figure S9 Linkage disequilibrium of European and Asian rapeseed populations

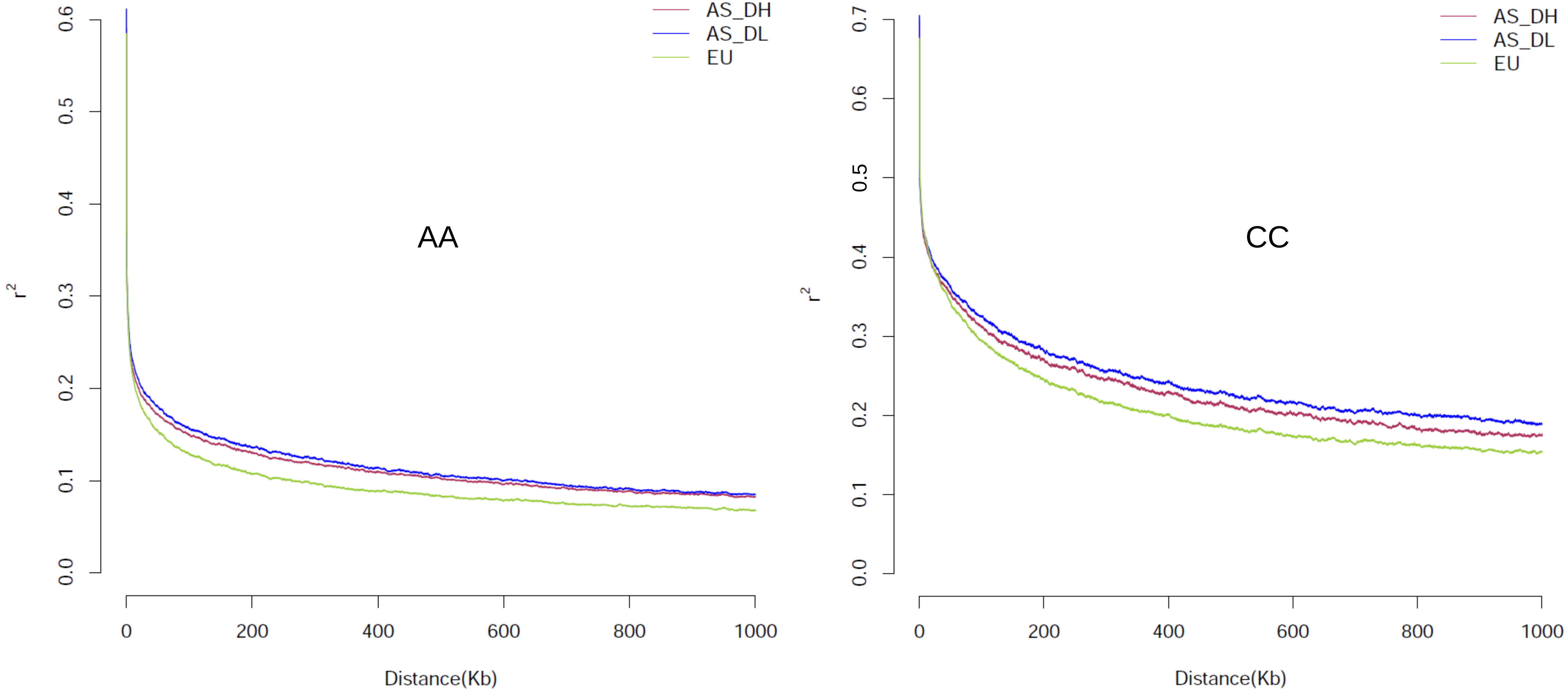

Figure S10 Demographic models of the origin of Asian rapeseeds

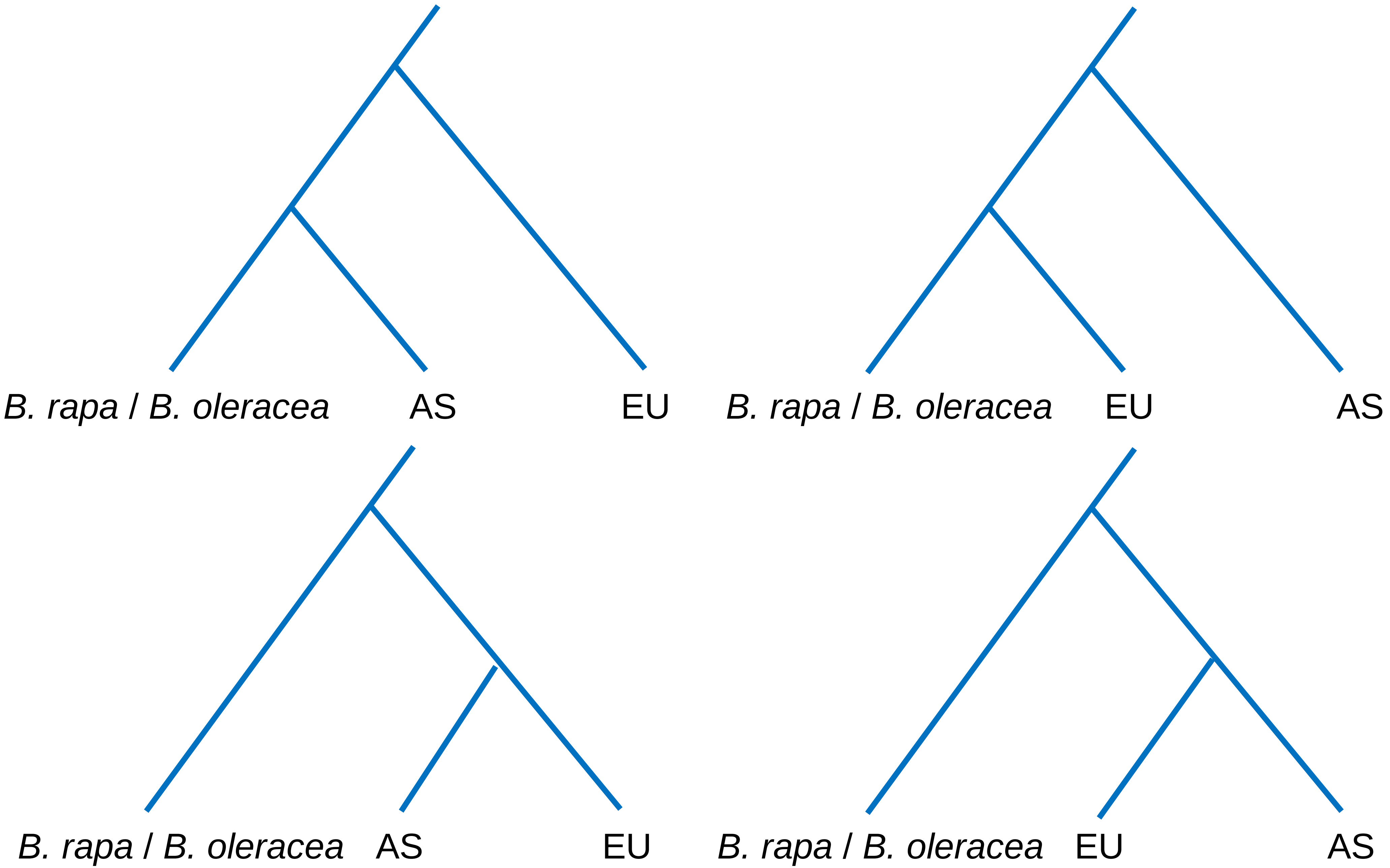

Figure S11 Demographic models of the origin of Asian double-low rapeseeds

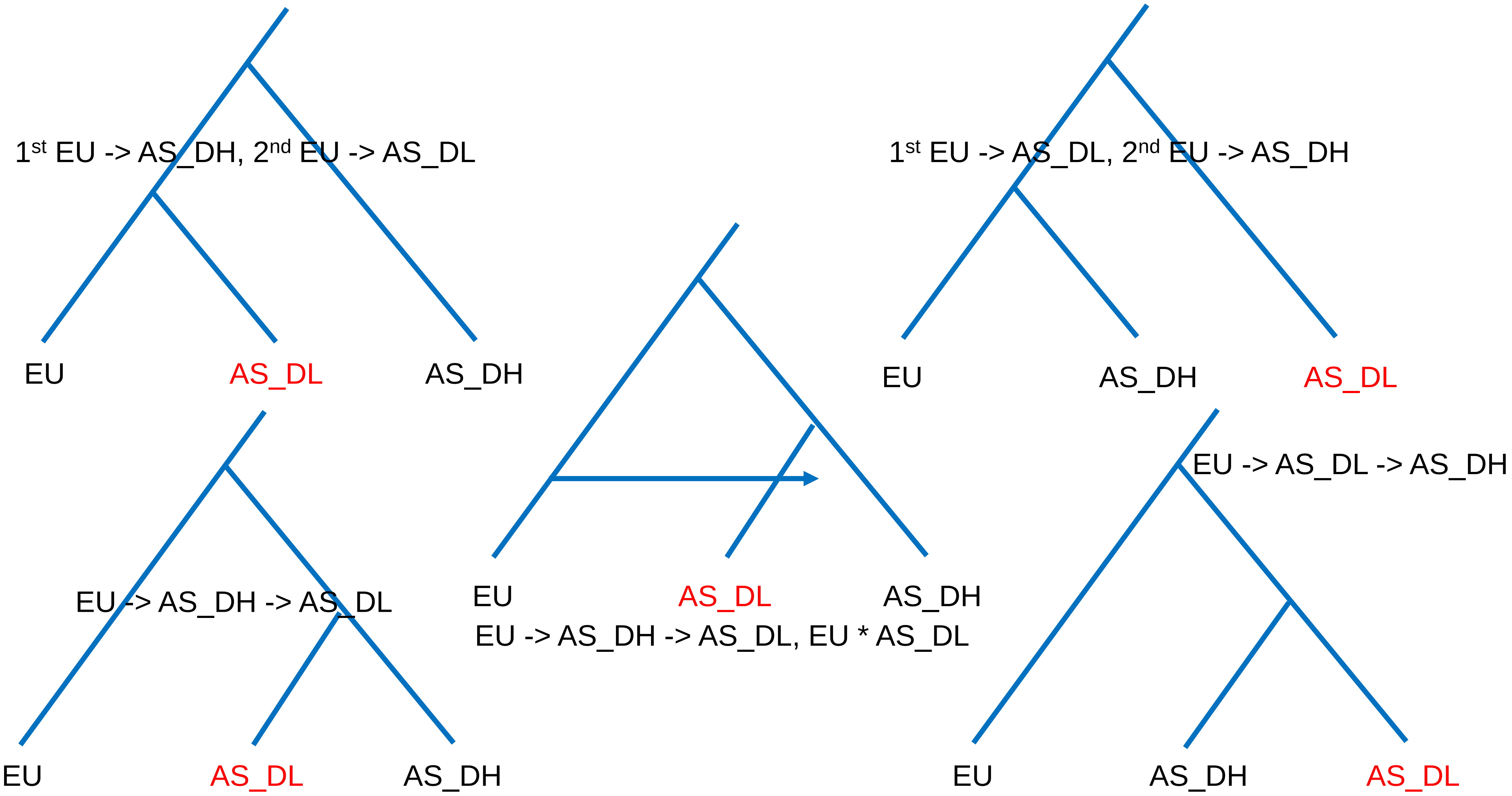

Figure S12 A genomic view of the read mapping information for *CDF2* homologue in the parental lines of NY7

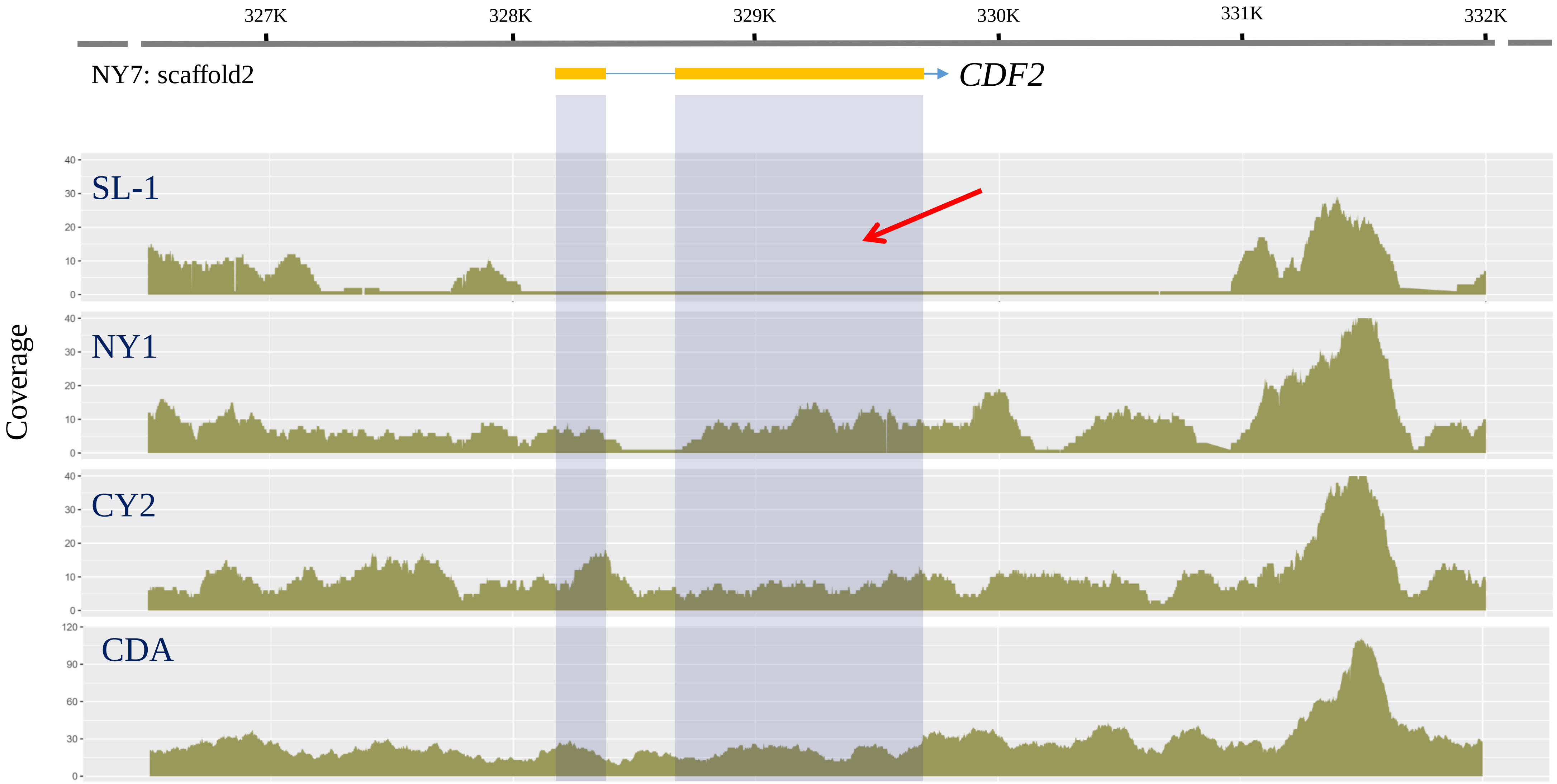

Figure S13 The frequency distribution of deleterious SNPs (dSNPs) and neutral intergenic SNPs (iSNPs) in (a) the NY7 pedigree (b) the Cn genome of the Asian and European populations

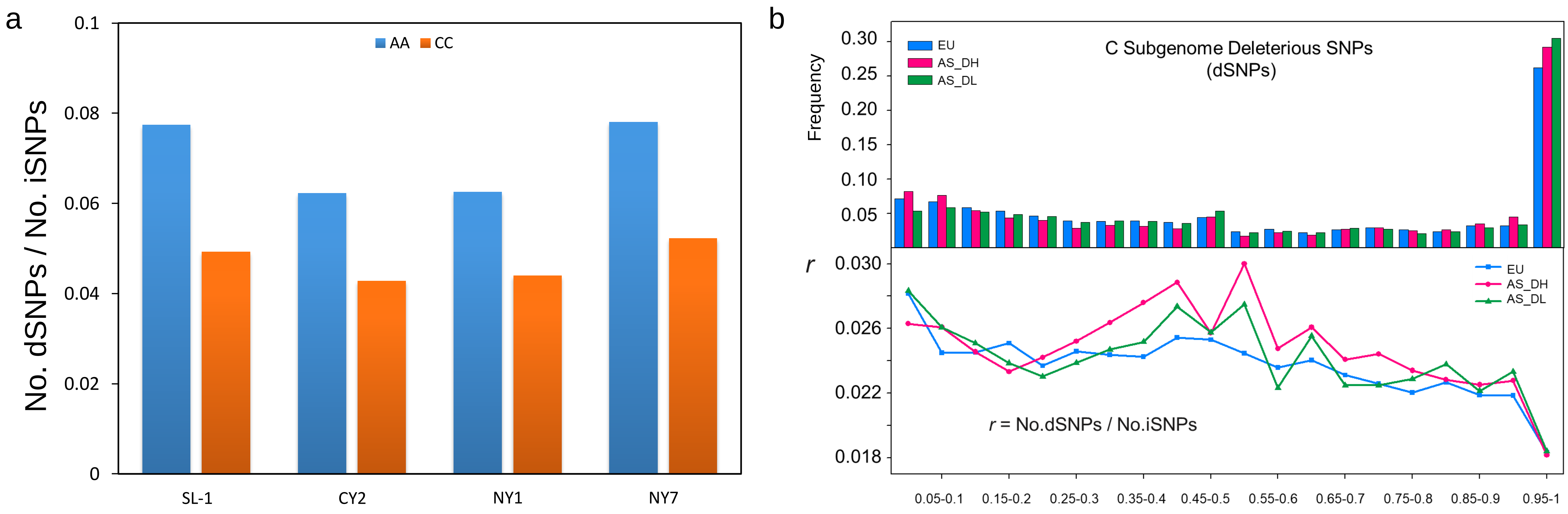

Figure S14 (a) Local ancestry inference of the introgression of *B. rapa* for Asian rapeseed. On the top of each box, the red signals represent the conserved blocks with introgression from *B. rapa*. (b) Local ancestry inference of the introgression of European lineage (blue) in Asian double-low cultivars

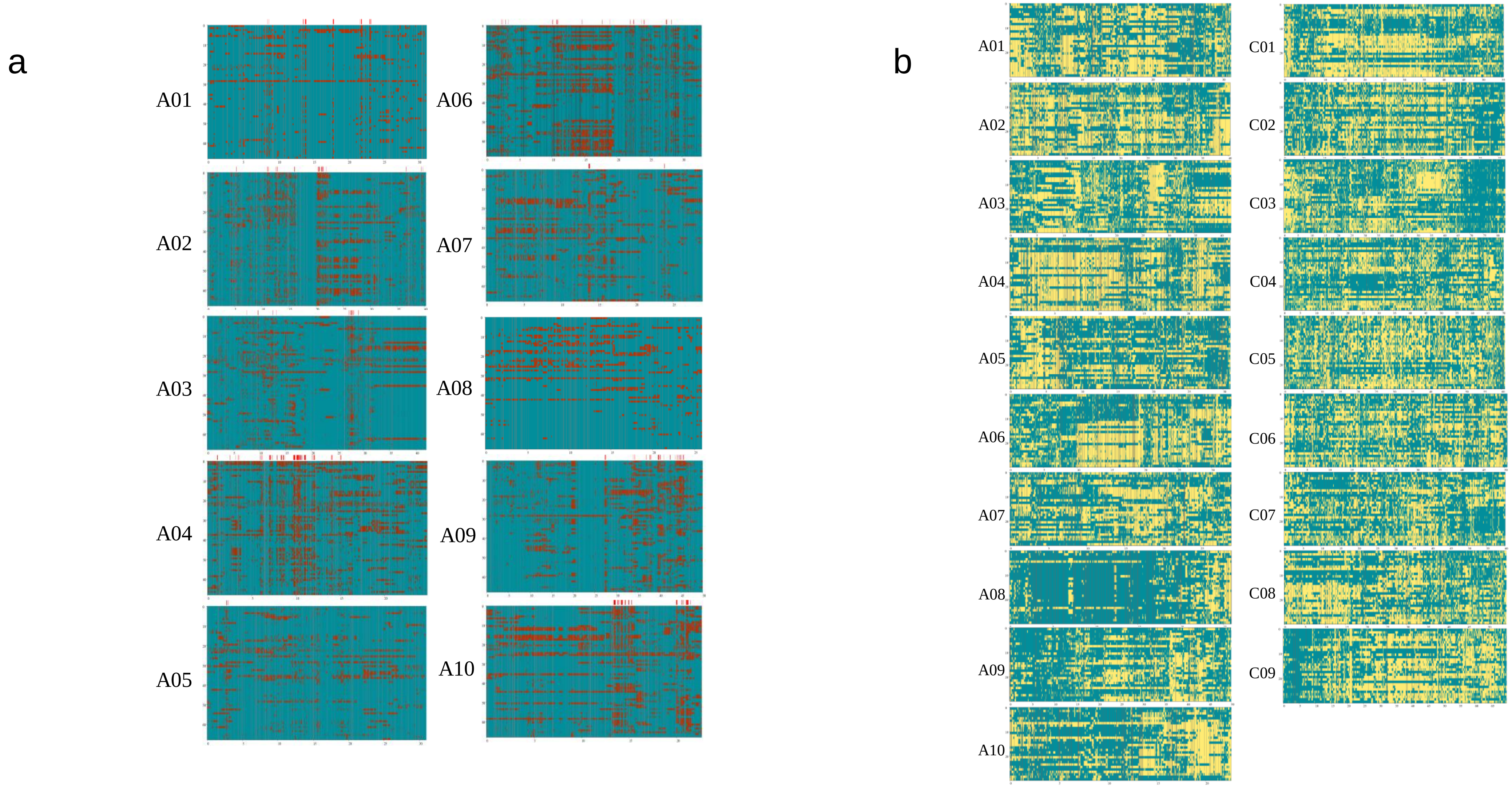

Figure S15 (a) A genomic view of NY1 reads in the homoeologous chromosome pair A01 and C01 with two diploid ancestors (*B. rapa* and *B. oleracea*) as references. (b) A genomic view of NY1 and CY2 reads in the homoeologous chromosome pair A09 and C09 with two diploid ancestors (*B. rapa* and *B. oleracea*) as references.

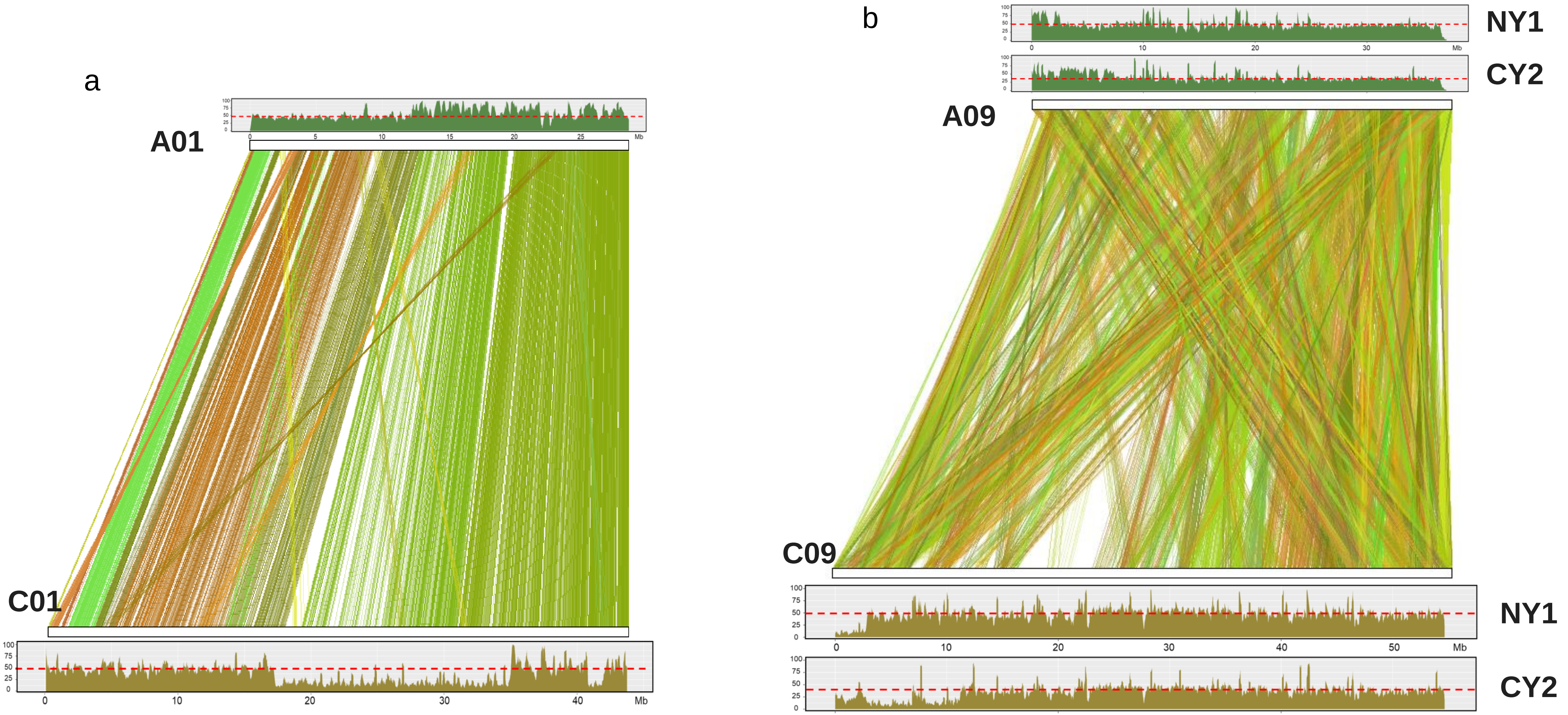

Figure S16 A summary of the origin and breeding history of Asian rapeseeds and the dynamic changes of genomic diversity and deleterious SNP (dSNP) accumulations in the two stages of the Asian local breeding history. The Asian genomes were significantly re-shaped by two genetic introgression events contributed by *B. rapa* and double-low European *B. napus*

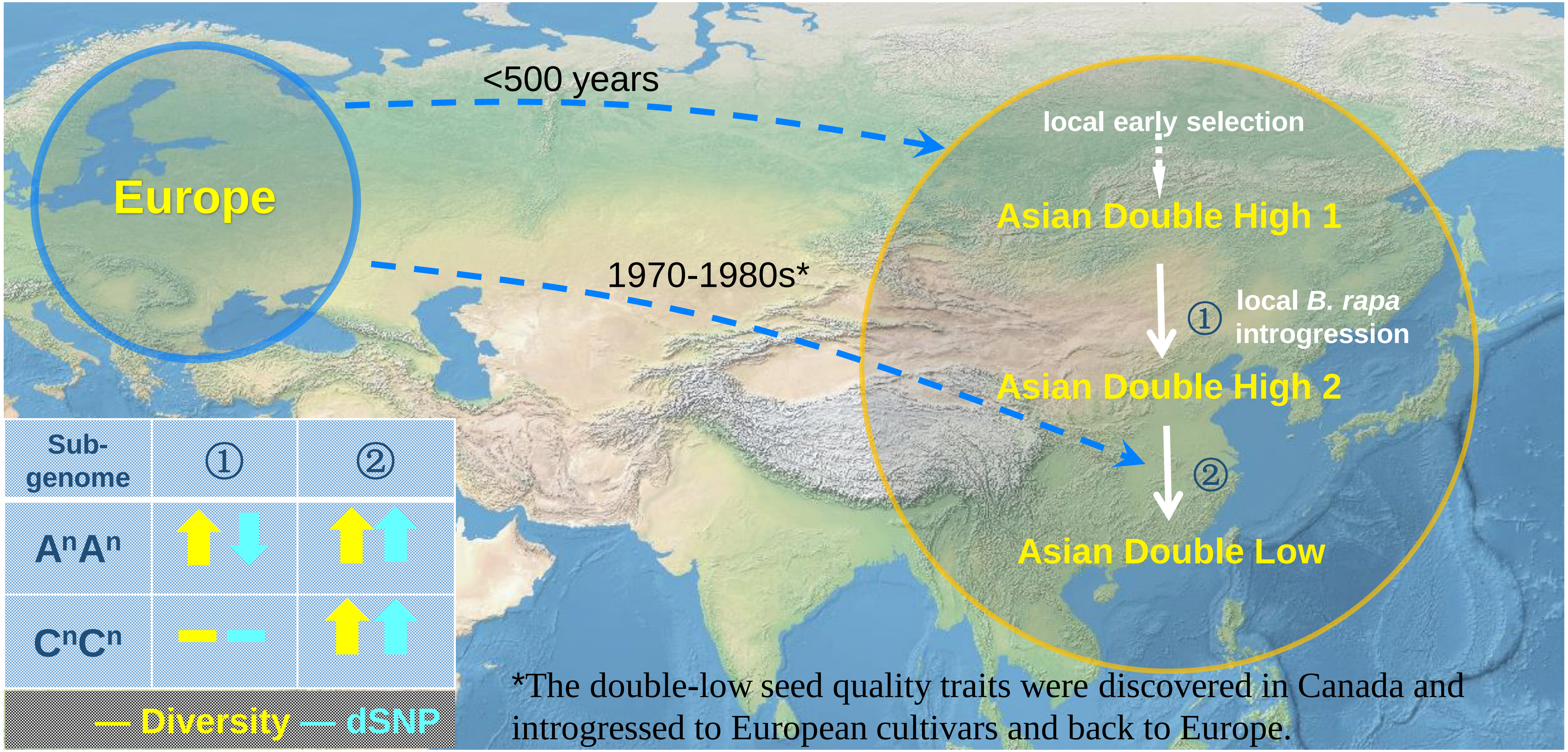

Supplement: Supplementary file 1 — Figure S1 Genomic survey of NY7 and its pedigree accessions with K‐mer distribution. Figure S2 The Hi‐C contact maps for the whole genome (left panel) and chrA03 (right panel) of NY7. Figure S3 The collinearity between each of the three B. napus genetic maps and the NY7 genome assembly. Figure S4 The pipeline for genome assembly and pseudo‐chromosome construction of NY7. Figure S5 The genomic synteny between NY7 and other de novo assemblies of B. napus and two diploid progenitors. a‐e present the synteny between NY7 and Darmor‐bzh, progenitor diploid B. rapa and B. oleracea, Tapidor reported by Bayer et al., 2017, Tapidor updated in this study, and ZS11, respectively; f presents the synteny between the two Tapidor assemblies, the one generated in this study and the one reported by Bayer et al., 2017. Figure S6 Demonstration of the QTL located in the genomic divergent region between the NY7 and Tapidor genomes. Genomic alignment view of chromosome C03 of the Tapidor assembly mapped to the NY7 reference. All QTL identified in C03 were labelled with ‘es.’ at the top of the rectangle. The arrow shows a QTL (es. C3‐3) region with present and absent sequence variation between Tapidor and NY7. Significantly high read coverage in the NY7 genome was found in the QTL (es. C3‐3) region. Figure S7 Phylogenetic trees for B. napus accessions based on SNPs found in both the A and C subgenomes. The EU, AS_DL, AS_DH represent the European rapeseed (B. napus), Asian rapeseed (B. napus) with double‐low and double‐high traits, respectively. Figure S8 Genetic diversity (π) of different chromosomes in different populations. EU and AS represent the European rapeseed (B. napus) and Asian rapeseed (B. napus), respectively. The EU, AS_DL, AS_DH represent European rapeseed, Asian rapeseed with double‐low and double‐high seed‐quality traits, respectively. Figure S9 Linkage disequilibrium of the European and Asian rapeseed populations. Figure S10 Demographic models used in assessing the origin [file PBI-17-1998-s001.pdf]
